# The hepatic transcriptome of the turkey poult (*Meleagris gallopavo*) is minimally altered by high inorganic dietary selenium

**DOI:** 10.1371/journal.pone.0232160

**Published:** 2020-05-07

**Authors:** Rachel M. Taylor, Kristelle M. Mendoza, Juan E. Abrahante, Kent M. Reed, Roger A. Sunde

**Affiliations:** 1 Department of Nutritional Sciences, University of Wisconsin, Madison, Wisconsin, United States of America; 2 Department of Veterinary and Biomedical Sciences, College of Veterinary Medicine, University of Minnesota, St. Paul, Minnesota, United States of America; 3 University of Minnesota Informatics Institute, University of Minnesota, Minneapolis, Minnesota, United States of America; University of Illinois, UNITED STATES

## Abstract

There is interest in supplementing animals and humans with selenium (Se) above Se-adequate levels, but the only good biomarker for toxicity is tissue Se. We targeted liver because turkeys fed 5 μg Se/g have hepatic Se concentrations 6-fold above Se-adequate (0.4 μg Se/g) levels without effects on growth or health. Our objectives were (i) to identify transcript biomarkers for high Se status, which in turn would (ii) suggest proteins and pathways used by animals to adapt to high Se. Turkey poults were fed 0, 0.025, 0.4, 0.75 and 1.0 μg Se/g diet in experiment 1, and fed 0.4, 2.0 and 5.0 μg Se/g in experiment 2, as selenite, and the full liver transcriptome determined by RNA-Seq. The major effect of Se-deficiency was to down-regulate expression of a subset of selenoprotein transcripts, with little significant effect on general transcript expression. In response to high Se intake (2 and 5 μg Se/g) relative to Se-adequate turkeys, there were only a limited number of significant differentially expressed transcripts, all with only relatively small fold-changes. No transcript showed a consistent pattern of altered expression in response to high Se intakes across the 1, 2 and 5 μg Se/g treatments, and there were no associated metabolic pathways and biological functions that were significant and consistently found with high Se supplementation. Gene set enrichment analysis also found no gene sets that were consistently altered by high-Se and supernutritional-Se. A comparison of differentially expressed transcript sets with high Se transcript sets identified in mice provided high Se (~3 μg Se/g) also failed to identify common differentially expressed transcript sets between these two species. Collectively, this study indicates that turkeys do not alter gene expression in the liver as a homeostatic mechanism to adapt to high Se.

## Introduction

Selenium (Se) was first identified as a toxic element for livestock in the 1930s [1], and then reported to be carcinogenic for rats [2], thus lumping Se as a poison with other elements like arsenic. In 1957, however, Se was found to be an essential nutrient for rats [3] and chickens [4], and soon after was shown to prevent a cardiomyopathy in humans called Keshan disease [5]. In 1973, Se was identified as an essential cofactor for glutathione peroxidase (GPX), establishing a biochemical role for Se as a required essential trace element [6]. Tissue levels of GPX and many other selenoenzymes fall dramatically in animals fed Se-deficient diets, reach a well-defined plateau above a breakpoint, and thus are good biomarkers for assessment of Se-deficient vs. Se-adequate status [7]. Using this approach, the minimum dietary Se requirement in turkey poults is 0.4 μg Se/g [8–10], which is 4-fold higher than minimum Se requirements in rats [11]. Thus tissue Se concentrations, signs of Se-specific disease, and selenoenzyme activities can be good biomarkers of Se status and Se deficiency.

Co-translational synthesis and insertion of the Se cofactor, selenocysteine, into GPX and other selenoproteins requires an in-frame UGA and a 3’UTR stemloop (the SECIS element) in the transcript to facilitate incorporation of selenocysteine at what otherwise would be a stop codon [12]. Using these transcript elements as a signature to screen annotated genomes for selenoproteins, 25 human, 24 rodent, and 24 avian selenoproteins–the entire selenoproteome–have been characterized [13–15]. No selenoenzyme activity, however, has been identified that increases substantially above levels in Se-adequate animals when diets are supplemented with Se at levels above the requirement [7].

In rats, there is no effect on growth in male rats fed 2 μg Se/g, but a 20% decrease in growth in rats fed 5 μg Se/g [16], whereas poultry appear less susceptible to Se toxicity. Chicks fed 1 and 5 μg Se/g for 4 wk show no effect on body weight as compared to chicks supplemented with Se-adequate diets [17,18], and the same is observed in turkey poults fed 1–5 μg Se/g [9,10,19]. In both avians and rats, higher dietary Se levels and longer exposures do result in toxicity [20–22]. In these species, blood and tissue Se concentrations increase above the Se requirement in a near linear fashion with increasing Se intake, but do not plateau; at 5 μg Se/g, rat kidney accumulates more Se than liver whereas in the turkey poult, the liver accumulates substantially more Se than the kidney [9–11,23–26].

Transcript levels have potential as biomarkers for trace element status. Homeostatic mechanisms are present for altering uptake/export transporters, mineral carriers, and/or storage forms for Zn, Fe and Cu [27–29] across the range from deficiency to excess. In Se deficiency, a subset of selenoprotein transcripts decrease dramatically in Se deficiency in rodents, and can be used as biomarkers for Se deficiency [26]. We initially thought that animals might up-regulate expression of selenoprotein transcripts upon supernutritional Se supplementation, but found that this did not occur in rats, mice, chickens, turkeys or *C*. *elegans* [10,16,18,30]. Characterization of the effect of up to 2 μg Se/g diet as selenite in rats resulted in fewer than 10 transcripts with significant differential gene expression. Feeding 5 μg Se/g diet, however, significantly altered the expression of 4% of the rat transcriptome suggesting that the resulting transcript set might contain more transcripts that those specifically responding to high Se status, as this level of Se supplementation was associated with a 20% decrease in growth [16]. In a similar study which provided mice with 1.6 μg Se/ml in the drinking water for 16 wk (equivalent to feeding ~3 μg Se/g diet), gene set enrichment analysis also identified a small set of 18 liver transcripts with differential expression p < 0.05 [31]. We hypothesized that the turkey, apparently more resistant to high Se, might be a better model to identify transcripts (potential biomarkers) associated with changes in gene expression related to adaptation to high Se status.

To assess the full turkey transcriptome for the effect of Se status from Se-deficient to high-Se, we conducted RNA-Seq analysis on liver mRNA from turkey poults fed 0, 0.025, 0.4, 0.75 and 1.0 μg Se/g diet in experiment 1, and fed 0.4, 2.0 and 5.0 μg Se/g in experiment 2. Our objectives were (i) to identify transcript biomarkers for high Se status, which in turn (ii) would suggest proteins and pathways used by animals to adapt to high Se. We targeted the liver because turkey poults fed 5 μg Se/g have hepatic Se concentrations 6-fold above levels in poults fed Se-adequate diet (0.4 μg Se/g) but without apparent effects on growth or health [10].

## Methods

### Reagents

Molecular biology reagents were purchased from Promega (Madison, WI, USA), Invitrogen (Carlsbad, CA, USA), or Sigma (St. Louis, MO, USA). All other chemicals were of molecular biology or reagent grade.

### Animals and diets

In both experiments 1 and 2, day-old male Nicholas White-derived turkey poults (kindly donated by Jennie-O Turkey Store, Barron, WI) were allocated randomly to treatment (5 poults/Se supplementation level) and housed in battery cages with raised wire floors and 24-hr lighting. The treatment and diets have been described in detail previously [8–10]. Briefly, the basal Se-deficient torula yeast-based diet (0.005 μg Se/g) was supplemented with 7.0% crystalline amino acids, including 0.93% methionine, to better match NRC recommendations for protein and amino acids [32]. Vitamin E (as all-rac-α-tocopherol acetate) was supplemented at 150 mg/kg (12.5X NRC requirement). In experiment 1, the basal diet was supplemented with 0, 0.025, 0.4, 0.75 or 1 μg Se/g diet as Na_2_SeO_3_. In experiment 2, the basal diet was supplemented with 0.4, 2 or 5 μg Se/g diet as Na_2_SeO_3_. The animal protocol was approved by the Research Animal Resources Committee at the University of Wisconsin-Madison (protocol no. A005368).

### Tissue Se and Selenoenzymes

Poults in both experiments were killed at 28 days by terminal CO_2_ overexposure followed by exsanguination. Entire livers were collected and immediately frozen at -80°C until analysis. Liver Se concentrations (n = 4) and selenoenzyme activities (n = 5) for plasma GPX3, and liver GPX1, GPX4, and thioredoxin reductase (TXNRD) were measured as described previously [8–10]. Data were analyzed by one-way ANOVA and variance equality was tested using Levene’s test for homogeneity of variances [33]. When the main effect of diet was significant, differences between means were assessed by Duncan’s multiple range test (p < 0.05) with Kramer’s modification for unequal class sizes when necessary [34].

### RNA isolation and high-throughput sequencing

Total RNA from liver tissues (n = 4/treatment) was isolated with TRIzol Reagent (Invitrogen, catalog no. 15596–026) following the manufacturer’s protocol. The RNA pellet was dissolved in 300 μL diethylpyrocarbonate-treated water and quantitated using a DeNovix DS-11+ spectrophotometer (DeNovix Inc., Wilmington, DE). Library preparation and sequencing were done at the University of Wisconsin-Madison Biotechnology Center. Samples were prepared for sequencing with the Illumina TruSeqv4 sample preparation kit, according to manufacturer’s instructions. Libraries were multiplexed and sequenced on an Illumina HiSeq 2500. At the University of Minnesota, the 125-bp paired-end reads (27.2 ± 1.97 M reads/sample) were trimmed to removed sequence adaptors and low-quality bases (resulting 189 bp average inset mean), and mapped using Bowtie (v2.2.4.0) to the turkey genome build 5.0 Annotation 102, as described previously [35], resulting in an average of 16,132 genes detected (min 15,685, max 16,567). The original RNA Seq data discussed in this publication have been deposited in NIH's NCBI’s Sequence Read Archive (SRA) and are accessible through SRA accession PRJNA614544 (available at https://www.ncbi.nlm.nih.gov/sra/PRJNA614544).

A number of the selenotranscripts in the NCBI turkey Annotation 102 are mis-annotated, mis-assembled, or missing. For the 24 selenoproteins plus selenophosphate synthase1 (SEPHS1, not a selenoprotein in the turkey), corrected full-length selenotranscript sequences were generated based on the turkey Annotation 102, combined with our cloned and sequenced turkey selenotranscripts [14] and with unplaced turkey genome scaffold sequences, to include the selenocysteine UGA codon, the SECIS element, and the full 5’ and 3’UTR when sequences were available, and then assembled into a pseudo genome and remapped. Briefly in Annotation 102, SELENOU (Selenoprotein U) is designated as FAM213A ID:100550701, SELENOP1 (Selenoprotein P1) is misassembled and portions designated as SELENOP ID:100547086 and as LOC104914865 ID:104914865, SELENOP2 is designated as LOC100546913 ID:100546913, TXNRD1 (Thioredoxin reductase 1) is designated as LOC100545667 ID: 100545667, and SELENOW (Selenoprotein W) is not present in Annotation 102. Many others are mis-annotated. Based on raw counts (≥20), 21 of the 24 turkey selenotranscripts were judged to be present in this mapping, with DIO3 (Deiodinase 3), GPX2, and SELENOH (Selenoprotein H) not present.

### Functional analysis

Principal component analysis (PCA) was performed with the R package FactoMineR [36] to identify and quantify variability in the data. Volcano plots were constructed to visualize the range of differential expression and significance across treatment groups. For differential expression analysis, pairwise comparisons between the Se-adequate control group (0.4 μg Se/g diet) and all other treatment groups were made in the R package DESeq2 [37], following the standard workflow. The false discovery rate (FDR) significance is designated as q.

Hierarchical cluster analysis was conducted, using fold-change values of differentially expressed (DE) transcripts (q < 0.1) in liver of poults fed graded levels of dietary Se (n = 4), as compared to Se-adequate (0.4 μg Se/g) poults, as generated by the likelihood ratio test (LRT) in DESeq2, subjected to centroid-linked Pearson correlation clustering implemented in Cluster 3.0 and visualized with Java Treeview [38].

### Pathway analysis and gene ontology analysis

NCBI homolgene website (https://www.ncbi.nlm.nih.gov/homologene) was used to identify mammalian homologs for turkey genes with DE transcripts q < 0.05. This resulted in gene sets containing 7, 12 and 57 characterized genes for low Se (0 and 0.025 μg Se/g), moderately high Se (1 μg Se/g), and high Se (2 and 5 μg Se/g) treatments, respectively. These gene sets were analyzed using Enrichr for Human WikiPathways 2019 and for Biological Process 2018 [39].

Gene Set Enrichment Analysis (GSEA) [40,41] was used to assess the effect of Se status on two pre-determined sets of genes (Molecular Signatures Database v. 7.0 (MSigDB), http://software.broadinstitute.org/gsea/msigdb/index.jsp): the Hallmark gene set of 50 well-defined biological states or processes; the KEGG gene set of 186 gene sets derived from the KEGG pathway database; plus an additional Selenoprotein gene set of 25 turkey selenogenes (24 selenoproteins plus selenophosphate synthetase-1 [SEPHS1]) [13]. For this analysis, the full turkey transcriptome was reduced to 11,612 human gene transcripts. For each experiment, each Se treatment was analyzed by comparison with the corresponding Se-adequate (0.4 μg Se/g) treatment, and the datasets trimmed to transcripts with at least one sample >14 counts, and then normalized prior to GSEA (4 replicates/treatment). An FDR (q) < 0.25 is considered to be significant [41]. In addition, GSEA was conducted using the total MSigDB gene ontology (GO) collection of 9996 gene sets for biological processes, cellular components, and molecular functions.

## Results

### Se status

The response of the liver transcriptome in turkey poults was studied in two experiments that fed graded levels of inorganic Se (as selenite) in diets supplemented with vitamin E at 12.5X the NRC requirement [32] so that direct effects of altered Se status could be studied without downstream secondary effects (disease) arising from physiological effects of Se deficiency or toxicity in combination with marginal vitamin E status. Characterizations of poult growth, selenoenzyme activities and selenoprotein transcript expression from experiment 1 [8,9] and experiment 2 [10] have been reported in detail previously. Briefly, there were no overt signs of impaired health due to Se supplementation levels and no significant effects on final body weight **(Table 1**). In 0 μg Se/g poults, liver Se fell to 6% of levels in Se-adequate poults (0.4 μg Se/g), and selenoenzyme activities for GPX1, GPX4, and TXNRD decreased to 3–15% of Se-adequate levels, showing that the poults fed the 0 μg Se/g diet were Se deficient. In experiment 2, 5 μg Se/g raised liver Se to 6X the level in Se-adequate poults, thus confirming that these poults received high Se diets. These high Se diets, however, did not significantly increase levels of selenoenzyme activity above Se-adequate levels (**Table 1**). Collectively, the biomarkers of Se status confirm that these dietary treatments resulted in animals that were of Se-deficient to high-Se status.

**Table 1:** Liver selenoenzyme activities and Se concentration in turkey poults fed graded levels of dietary Se.

Expt	Se treatment	Final weight	Se conc.	GPX1 activity	GPX4 activity	TXNRD activity
	(μg/g)	(g)	(μg/g wet wt)	(EU/g)	(EU/g)	(EU/g)
Expt 1	0	526 ± 77*	0.38 ± 0.06^d^	3.2 ± 1.3^b^	4.4 ± 0.4^d^	0.41 ± 0.17^c^
	0.025	569 ± 67	1.12 ± 0.18^d^	4.6 ± 1.8^b^	8.3 ± 1.0^d^	1.31 ± 0.12^c^
	0.4	717 ± 113	6.03 ± 0.11^c^	95.1 ± 11.0^a^	73.0 ± 5.1^a^	2.70 ± 0.18^b^
	0.75	765 ± 76	8.55 ± 0.60^c^	100.2 ± 10.2^a^	64.4 ± 4.1^ab^	2.79 ± 0.28^b^
	1	647 ± 158	8.40 ± 0.18^c^	90.9 ± 20.0^a^	57.9 ± 8.2^b^	2.73 ± 0.08^b^
Expt 2	0.4	742 ± 61	5.37 ± 0.48^c^	73.4 ± 10.1^a^	32.7 ± 3.9^c^	3.08 ± 0.24^ab^
	2	569 ± 50	12.53 ± 0.26^b^	92.8 ± 17.1^a^	39.4 ± 5.8^c^	2.97 ± 0.28^ab^
	5	588 ± 70	31.85 ± 3.12^a^	103.9 ± 8.4^a^	43.7 ± 2.4^bc^	3.48 ± 0.19^a^
	ANOVA p-value	0.366	4.61E-11	4.37E-06	2.34E-10	7.05E-12

*Values are mean ± SEM (n = 4 for Se; n = 5 for other biomarkers). Values not sharing a common letter are significantly different (p< 0.05).

### PCA plots

**Fig 1:**
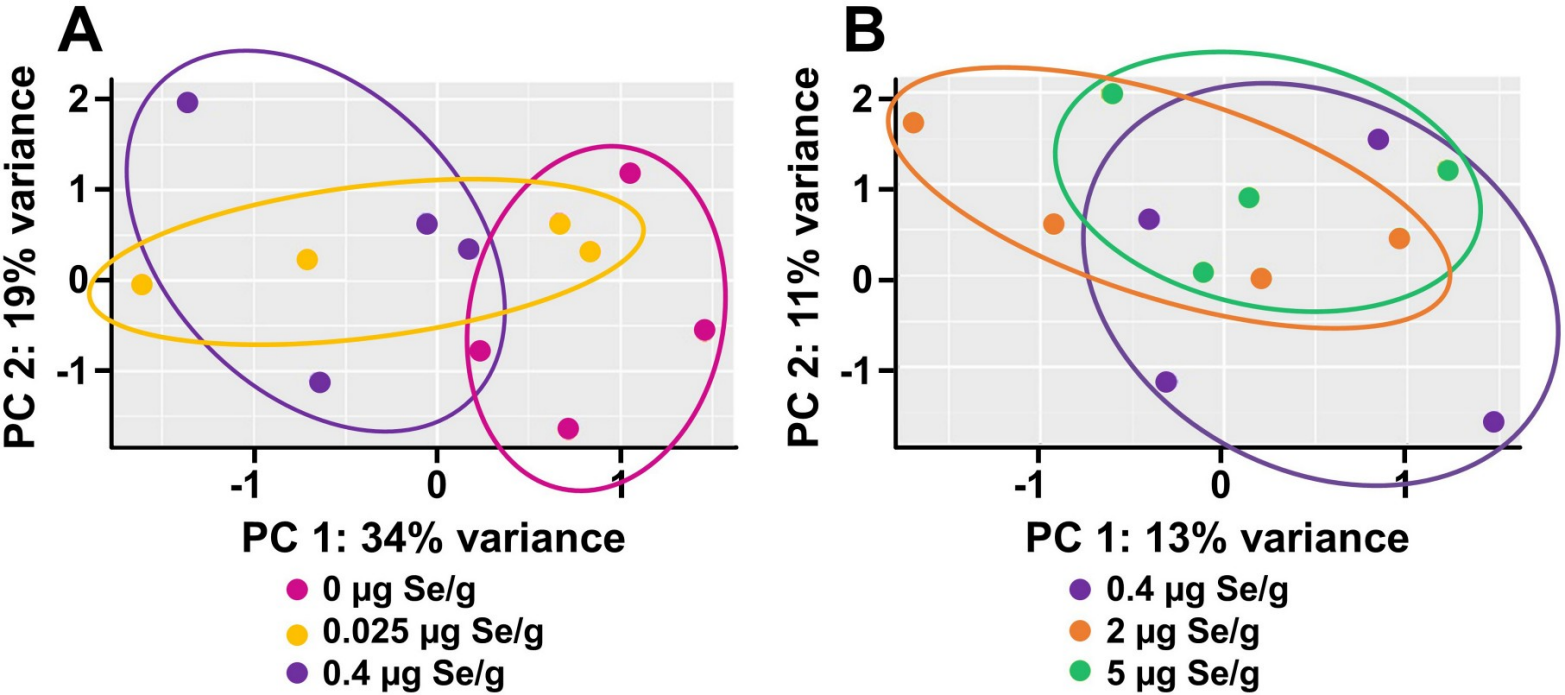
Principal component analysis (PCA) of liver transcripts (n = 17,100) from poults supplemented with graded levels of Se for 28 d. (A) Analysis of low-Se treatments in experiment 1; (B) Analysis of high Se treatments in experiment 2.

PCA was performed either using all ~17,100 transcripts expressed at or above the level of detection in each sample from both experiments or using the 500 highest-expressed transcripts in each sample; no significant clustering was observed between treatment groups. When PCA was conducted for the effect of low Se by comparing 0 and 0.025 vs. 0.4 μg Se/g groups in experiment 1, poults supplemented with 0 μg Se/g diet tended to cluster together and PCA accounted for 53% of the variation (**Fig 1A**). When PCA was conducted for the effect of high Se by comparing 2 and 5 vs. 0.4 μg Se/g groups in experiment 2, there was no notable clustering of samples within treatment groups and PCA could account for only 24% of the variation (**Fig 1B**). These PCA analyses indicate that high Se did not exert major effects on general transcript expression in these studies.

### Volcano plots

**Fig 2:**
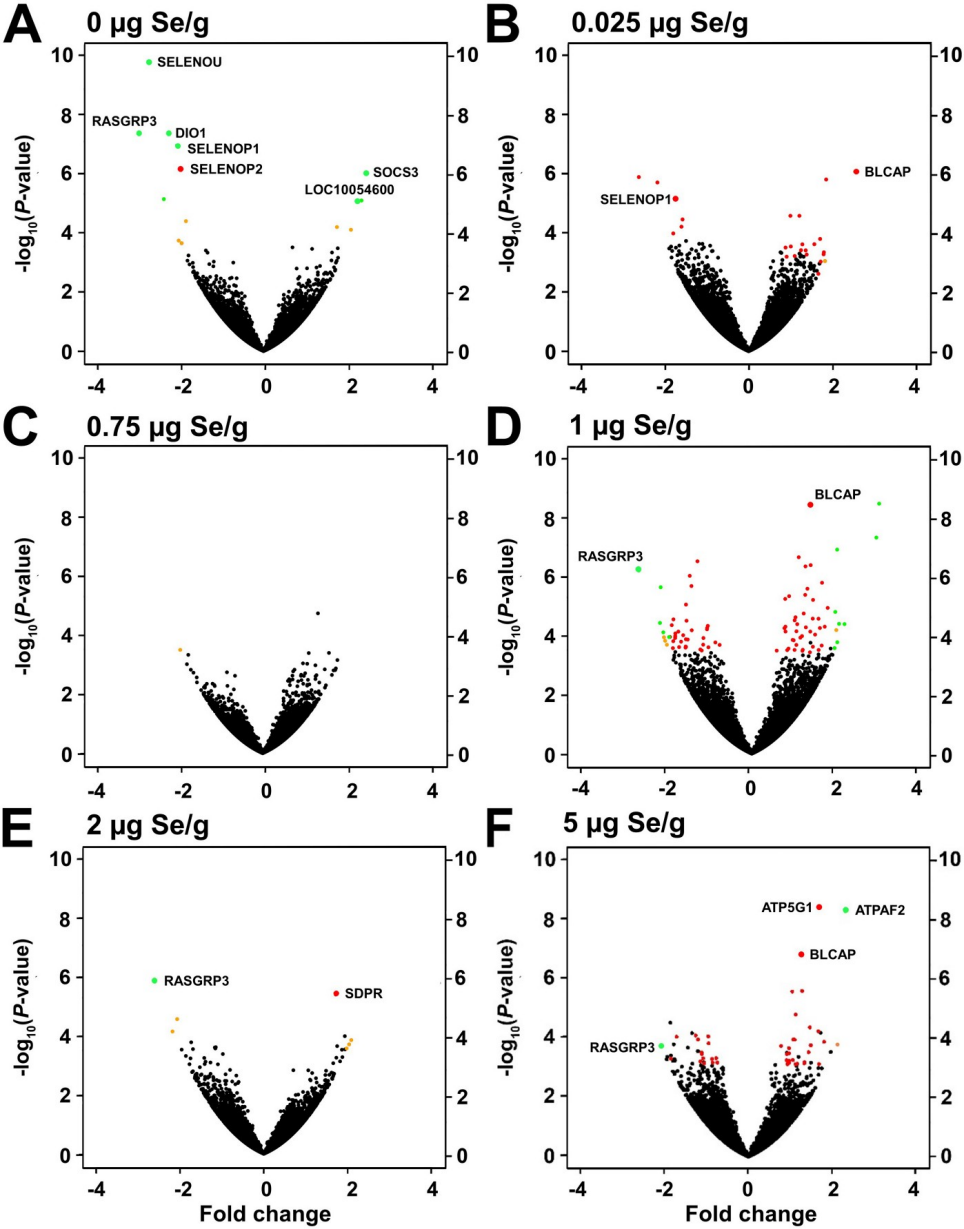
Volcano plots depicting the effect of dietary Se on differential expression of liver transcripts (n = 17,100) in poults supplemented with graded levels of Se for 28 d. Plotted are log_10_(unadjusted p-value) vs. fold change (FC). (A) 0 vs 0.4 μg/g, (B) 0.025 vs 0.4 μg/g, (C) 0.75 vs 0.4 μg/g, (D) 1 vs 0.4 μg/g, (E) 2 vs 0.4 μg/g, (F) 5 vs 0.4 μg/g. Values are means (n = 4). Orange, FC ≥ |±2| only; Red, q ≤ 0.1 only; Green, q ≤ 0.1 and FC ≥ |±2|; Black, neither condition satisfied.

For each Se treatment, volcano plots were constructed to show average fold changes and significance relative to the Se-adequate average for that experiment (**Fig 2**). In Se-deficient poults, 10 transcripts were significantly DE, including 4 down-regulated selenotranscripts: DIO1, SELENOP1, SELENOP2, and SELENOU (FAM213A) (**Fig 2A**). In poults supplemented with 0.025 μg/g, 23 transcripts were DE, including SELENOP1 (**Fig 2B**). Supplementing with 0.75, 1 and 2 μg/g resulted in 0, 218 and 2 DE transcripts, respectively (**Fig 2C–2E**). High-Se poults had 45 DE liver transcripts (**Fig 2F**), demonstrating that increasing Se supplementation did not increasingly enlarge the total number of DE transcripts. In addition, few transcript in any treatment had fold-changes >|±2|.

### Differential expression

#### Se-deficient and low Se

Pairwise analysis of the 0 vs. 0.4 μg Se/g treatments found just 10 DE transcripts with q < 0.05, including 4 down-regulated selenotranscripts: SELENOU, DIO1, SELENOP1 and SELENOP2. Non-selenoprotein transcripts HSPB11, RASGRP3, and SGTA were also significantly down-regulated by Se deficiency; SOCS3 and 2 uncharacterized transcripts were significantly up-regulated (**Table 2**). HSPB11 is a heat shock protein family B (small) member 11, RASGRP3 is a RAS guanyl releasing protein 3, SGTA is a small glutamine rich tetratricopeptide repeat containing alpha, SOCS3 is a suppressor of cytokine signaling 3, LOC104909734 is a sulfotransferase family cytosolic 2B member 1-like, and LOC100545600 is a C-C motif chemokine 3-like protein. The function and NCBI Gene ID for all significant DE transcripts found in the 0, 0.025, 2, and 5 μg Se/g comparisons are given in **S1 Table**. Analysis of the 0.025 vs. 0.4 μg Se/g treatments found just 2 DE transcripts with q < 0.05; this included significant down-regulation of SELENOP1 expression and up-regulation of BLCAP expression. BLCAP is a bladder cancer-associated protein. For 0 or 0.025 μg Se/g, 3 additional selenoprotein transcripts, TXNRD3, GPX4, and GPX1, had DE with unadjusted p < 0.05 but q > 0.1 (**Table 2**). The only characterized transcripts up-regulated by low Se were SOCS3 and BLCAP by 0 and 0.025 μg Se/g, respectively. SOCS3 is a suppressor of cytokine signaling 3. Clearly low Se status targeted a subset of selenoprotein transcripts for down-regulation, but there was little additional impact of Se deficiency on the transcriptome.

**Table 2:** Transcripts DE by 0 and 0.025 μg Se/g vs. Se-adequate (Exp 1)*.

Symbol	Gene ID	Fold	p-value	q-value	0.75	1	2	5
**0 μg Se/g Differentially Expressed Transcripts**					μg Se/g	μg Se/g	μg Se/g	μg Se/g
HSPB11	100543060	-2.52	5.02E-12	8.21E-08				
SELENOU	100550701	-2.62	1.64E-10	1.34E-06				
RASGRP3	100539029	-2.84	4.32E-08	0.0002		↓	↓	
DIO1	100546412	-2.21	4.43E-08	0.0002				
SELENOP1	100547086	-2.07	1.12E-07	0.0004				
SELENOP2	100546913	-2.00	6.59E-07	0.0018				
LOC104909734	104909734	2.36	9.30E-07	0.0022				
SGTA	100543804	-2.31	7.27E-06	0.0132				
SOCS3	100547594	2.28	8.07E-06	0.0132				
LOC100545600	100545600	2.21	8.08E-06	0.0132				
TXNRD3	100539983	-1.60	0.0005	0.3657				
GPX4	100379155	-1.89	0.0009	0.5592				
**0.025 μg Se/g Differentially Expressed Transcripts**								
SELENOP1	100547086	-1.85	7.47E-06	0.0138				
BLCAP	100547906	1.42	2.68E-05	0.0248		↑		↑
SELENOU	100550701	-1.66	0.0007	0.0956				
GPX1	100379156	-1.47	0.0033	0.1429				

*Fold changes, p-value, and FDR q-value for DE transcripts (q < 0.05) by 0 and 0.025 vs. 0.4 μg Se/g. Also listed are selected additional selenotranscripts with q >0.05. Arrows in columns (on right) indicate direction of fold-change for transcripts that were also DE (q < 0.05) by 0.75, 1, 2, and 5 vs 0.4 μg Se/g

#### High Se

Pairwise comparison of high dietary Se supplementation at 2 and 5 μg Se/g in experiment 2 only significantly altered gene expression for 2 and 14 transcripts, respectively; all changes were < |±2.5| fold, and there were no transcripts that overlapped between these two sets (**Table 3**). Moderate high Se (2 μg Se/g) significantly up-regulated SDPR, a serum deprivation response factor, and down-regulated RASGRP3, which was also one of the non-selenoprotein transcripts significantly down-regulated by Se deficiency. Dietary Se at 5 μg Se/g significantly up-regulated expression of 10 transcripts and down-regulated 4 transcripts; all but one change was < |±2.0| fold, and no selenotranscripts were significantly regulated. Included in up-regulated transcripts were genes for ATP synthases, kinases, a phosphatidylethanolamine binding protein, a mitochondrial CytC oxidase, and BLCAP which was also up-regulated by 0.025 but not 0 μg Se/g (**Tables 2 and 3**).

**Table 3:** Transcripts DE by 2.0 and 5.0 μg Se/g vs. Se-adequate (Exp 2)*.

Symbol	Gene ID	Fold	p-value	q-value	0	0.025	0.75	1	2	5
**2,0 μg Se/g Differentially Expressed Transcripts**					μg Se/g	μg Se/g	μg Se/g	μg Se/g	μg Se/g	μg Se/g
RASGRP3	100539029	-2.50	1.51E-06	2.57E-02	↓			↓	↓	
SDPR	100545738	1.83	4.11E-06	3.50E-02					↑	
SELENOI	100548385	-1.44	8.75E-03	0.9530						
SEPHS1	100550629	-1.36	2.58E-02	1.0000						
**5.0 μg Se/g Differentially Expressed Transcripts**					** **	** **	** **	** **	** **	** **
ATP5G1	100548571	1.78	3.79E-09	0.0000				↑		↑
ATPAF2	100544773	2.21	5.19E-09	0.0000						↑
BLCAP	100547906	1.55	1.59E-07	0.0003		↑		↑		↑
LOC100545745	100545745	1.55	2.60E-06	0.0032						↑
RNH1	100548440	1.43	2.71E-06	0.0032				↑		↑
DAD1	100547759	1.47	0.0000	0.0158				↑		↑
RPS27A	100542056	1.65	0.0000	0.0365						↑
BRI3	104913503	1.77	5.92E-05	0.0431						↑
CCND1	100539759	-1.54	8.20E-05	0.0472						↓
CHCHD2	100550053	1.44	1.12E-04	0.0472						↑
PDPK1	100541724	-1.51	0.0001	0.0472				↓		↓
CDC42BPB	100550337	-1.39	0.0001	0.0472						↓
PEBP1	100545499	1.46	0.0001	0.0472				↑		↓
LOC104912775	104912775	-1.80	9.09E-05	0.0472						↓
SEPHS1	100550629	1.41	0.0232	1.0000						

*Fold changes, p-value, and FDR q-value for DE transcripts (q < 0.05) by 2 and 5 vs. 0.4 μg Se/g. Also listed are selected additional selenotranscripts with q >0.05. Arrows in columns (on right) indicate direction of fold-change for transcripts that were also DE (q < 0.05) by 0, 0.025, 0.75, 1, 2, and 5 vs 0.4 μg Se/g

#### Supernutritional Se

Pairwise comparison of 0.75 μg Se/g with Se-adequate treatment in experiment 1 did not result in any DE transcripts. This indicates that this level of supernutritional Se supplementation had little or no effect on the general transcriptome (**S2 Table**).

Supplementation with 1 μg Se/g in experiment 1 resulted in the largest set of DE transcripts (**S2 Table**): 50 up- and 38 down-regulated. These transcripts included BLCAP which was also up regulated by 0.025 but not 0 μg Se/g, and RASGRP3 which was also down-regulated by 0 but not 0.025 μg Se/g. These U-shaped and inverted-U-shaped responses show that expression of these transcripts was not changed solely in response to increased Se status. The set of 88 DE transcripts for 1 μg Se/g, however, did include 6 transcripts which were also significantly up-regulated and 1 transcript that was also significantly down-regulated by 5 μg Se/g, but not by 2 μg Se/g or by low Se treatment (**Table 3**). Amongst these 7 transcripts with significant DE by 1 and 5 μg Se/g, no transcript also had DE with unadjusted p < 0.05 for 2 μg Se/g, indicating that changes in DE of these transcripts were not uniquely associated with high Se status.

### Hierarchical cluster analysis

**Fig 3:**
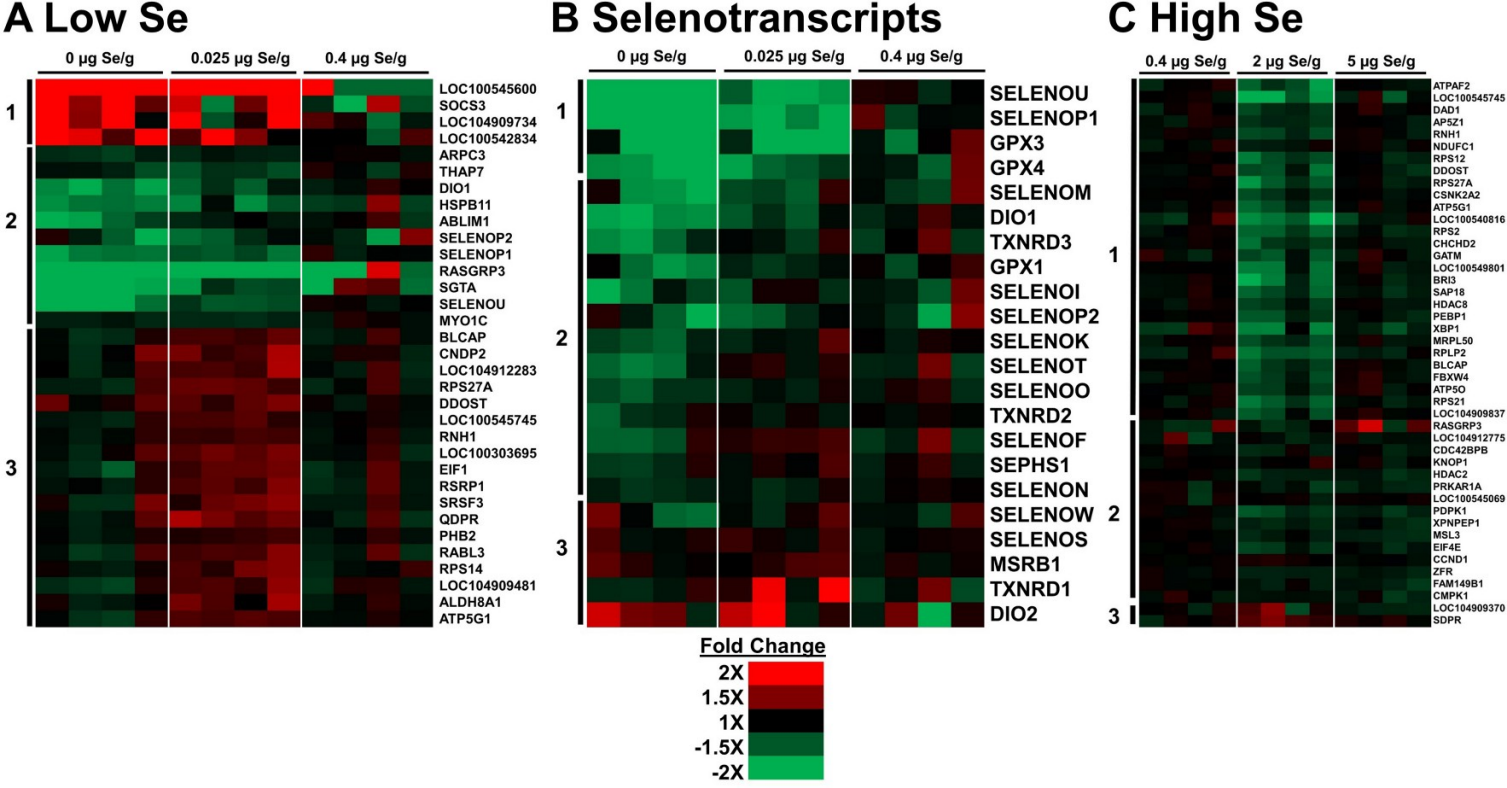
Hierarchical cluster analysis of significant DE transcripts (q < 0.1 in one or more treatments). Values (n = 4/treatment) are fold changes relative to the mean of Se-adequate (0.4 μg Se/g) poults. (A) Analysis of low-Se treatments in study 1 (n = 33); (B) Analysis of remapped selenotranscripts of low-Se treatments in study 1 (n = 22); (C) Analysis of high Se treatments in study 2 (n = 45). Vertical bars show major clusters.

The overall pattern of regulation by Se-deficient and low-Se status was further examined by hierarchical cluster analysis on an expanded set of 33 DE transcripts with q < 0.1 (**Fig 3A**). Across the Se treatments (0–0.4 μg Se/g), there was a cluster of down-regulated transcript that included four selenotranscripts (SELENOP1, SELENOP2, DIO1, SELENOU), and a cluster of four up-regulated transcripts. This analysis indicated that almost half of these transcripts showed near 2-fold regulation, up or down, by 0 μg Se/g vs. Se-adequate 0.4. In addition, a third large cluster of transcripts showed inverted U-shaped expression with levels at 0.025 μg Se/g higher than at 0 or 0.4 μg Se/g (**Fig 3A**). When the remapped selenoprotein transcripts were subjected to cluster analysis (**Fig 3B**), a cluster of four selenotranscripts were down-regulated by both 0 and 0.025 μg Se/g, and a cluster of 14 additional selenotranscripts were down-regulated only by 0 μg Se/g, similar to our previous reports [8–10].

Cluster analysis of the high Se treatments revealed 3 clusters, with only modest fold-changes relative to Se-adequate treatment (**Fig 3C**). The largest cluster showed down-regulation of transcripts by 2 μg Se/g but not by 5 or 0.4 μg Se/g–an inverted U-shaped response. A second cluster showed down-regulation by 2 and 5 μg Se/g vs 0.4 μg Se/g. Lastly, a small cluster of 2 transcripts showed a U-shaped response, i.e. up-regulation by 2 μg Se/g. Thus, there was not a consistent pattern of regulation in high Se and few of these transcripts appeared to be good potential biomarkers for high Se status.

### Pathway and gene ontology analysis

Transcripts sets with significant DE associated with low Se, high Se, and supernutritional Se were used to identify homologous human genes that were assessed for enriched pathways and biological functions (Enrichr) [39]. For the low Se set containing 7 humanized genes, selenoprotein metabolism and Se nutrient network were nearly significant, with 0.05 < q < 0.10 (**S3 Table**), but with no significant enrichment of biological processes (**S4 Table**). Analysis of high Se treatment in experiment 2 with 12 humanized genes revealed no significant pathway enrichment (all q > 0.2) or biological function enrichment (q = 1). Lastly, moderate high Se at 1 μg Se/g, with 57 DE orthologous genes, had significant enrichment in proteins associated with cytosolic ribosomal proteins (q = 0.003) and nearly significant enrichment of regulation of glycolysis (q = 0.052), with significant biological functions including protein synthesis and targeting, nuclear transcribed RNA processing, and response to EGF stimulation; none of these biological functions were significant for high Se (2 and 5 μg Se/g) treatments.

### GSEA

Gene set enrichment analysis (GSEA) is an approach that evaluates transcript expression data at the level of gene sets to detect changes in pathways and biological processes that are coordinated at a more subtle level than found by DE analysis of individual genes [40,41]. In a recent report, this approach identified three significant pathways (q < 0.05) in liver that had increased DE of transcripts in mice supplemented with high Se; these were Hallmark gene sets for cholesterol homeostasis, pancreatic β-cell signaling, and fatty acid metabolism [31].

**Fig 4:**
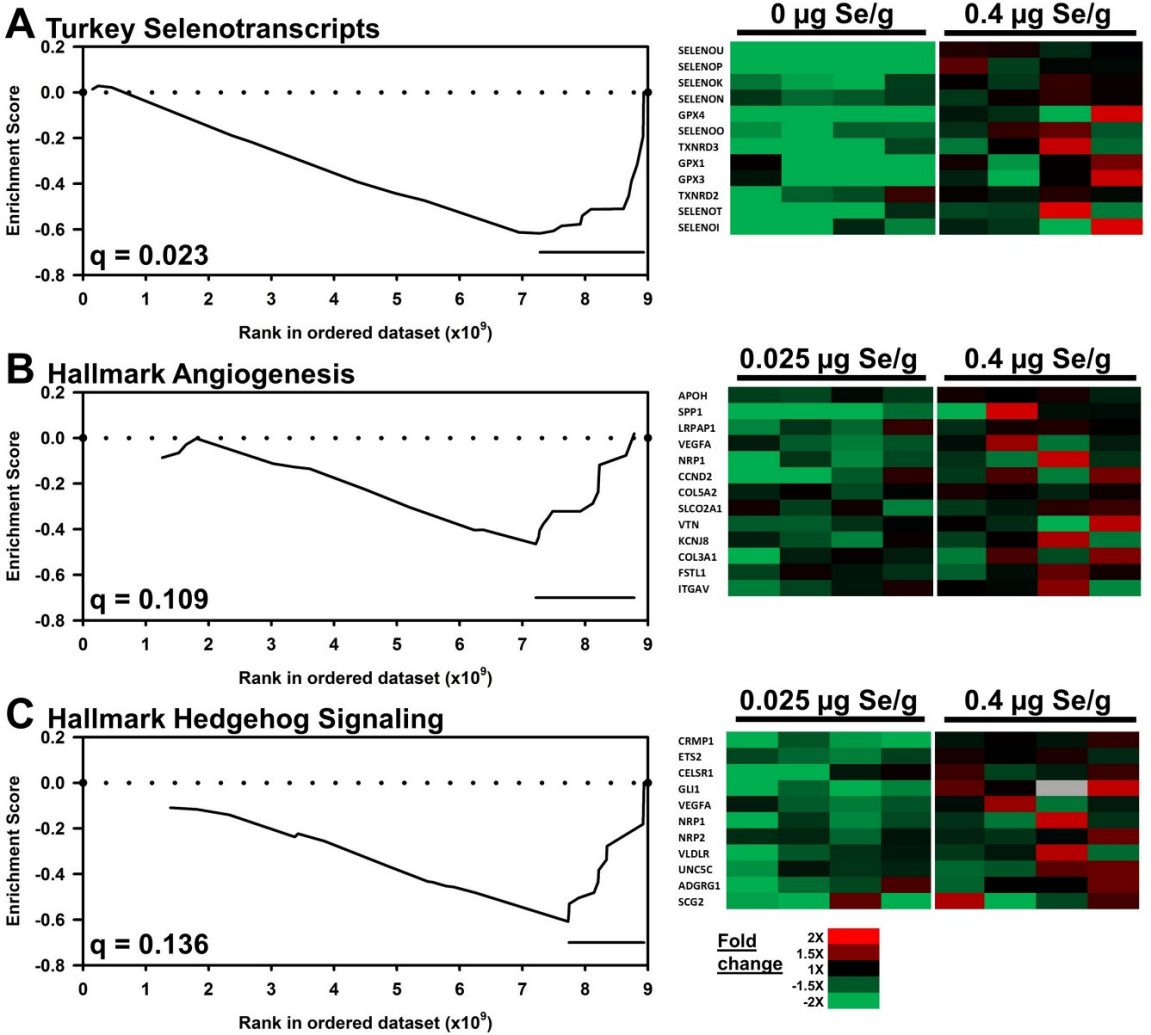
GSEA of liver transcripts in turkeys fed Se-deficient vs Se-adequate diet. Shown are resulting enrichment plots (left) and fold-change heat maps (right) for resulting leading-edge genes. (A) Turkey selenogene set; (B) Hallmark angiogenesis gene set; (C) Hallmark hedgehog signaling gene set. Enrichment score is plotted vs. the rank in the ordered dataset of 11,612 humanized turkey transcripts (trimmed to include >14 counts, and normalized). Bars mark the leading-edge genes shown in the corresponding heat map. FDR q value shown for each plot.

GSEA with the turkey selenoprotein gene set (22 of 25 were present) found that the selenoprotein gene set was significantly down-regulated (q = 0.023) by 0 vs. 0.4 μg Se/g supplementation, with 13 transcripts significantly contributing to the leading edge (**Fig 4A**). GSEA, however, did not find significant regulation of the turkey selenoprotein gene set by 0.025 vs. 0.4 μg Se/g (q = 0.454). These analyses affirm the results as assessed by individual DE analysis as well as previous analysis by qPCR [8,10], and demonstrate the effectiveness of the GSEA approach for this turkey RNA Seq dataset.

Using the full Se-deficient turkey liver transcriptome, however, GSEA identified no pathways that were significantly increased (all q > 0.9) or significantly decreased (all q > 0.4) when comparing Se-deficient (0 μg Se/g) vs. Se-adequate (0.4 μg Se/g) transcripts amongst the 50 Hallmark gene sets (**Table 4**). For low-Se (0.025 μg Se/g) vs. Se-adequate (0.4 μg Se/g), there also were no significantly increased Hallmark transcript pathways (all q > 0.3); two Hallmark pathways (angiogenesis and hedgehog signaling) were significantly decreased (q = 0.109 and 0.136, respectively) by low-Se treatment (**Fig 4B and 4C; Table 4**). Analyses using the set of 186 KEGG gene sets yielded similar results for up-regulated transcripts (Se-deficient vs Se-adequate, all q > 0.9); there were two KEGG pathways (pentose phosphate and ether lipid metabolism) significantly increased (q = 0.234 and 0.249, respectively) by 0.025 μg Se/g treatment (**S5 Table**). GSEA identified 12 KEGG pathways significantly decreased by Se-deficiency (q < 0.25), but no pathways significantly decreased by 0.025 μg Se/g (all q > 0.3). This application of GSEA was readily able to detect altered liver selenotranscript expression in Se deficiency even in turkeys with good vitamin E supplementation, but there were almost no Hallmark gene sets significantly affected by Se deficiency, and only a few KEGG gene sets were significantly regulated by low-Se; there were no gene sets in either group significantly regulated by both Se-deficiency and low-Se. What about high Se?

**Table 4:** GSEA Se-deficient and low-Se most significant Hallmark gene sets^a^.

**Down-Regulated States or Processes**	**0 μg Se/g**				**0.025 μg Se/g**			
** **	**No**	**ES**	**p-value**	**q-value**	**No**	**ES**	**p-value**	**q-value**
ANGIOGENESIS	23	-0.41	0.162	0.467	24	-0.50	0.030	0.109
HEDGEHOG SIGNALING	22	-0.40	0.468	0.466	23	-0.65	0.000	0.136
EPITHELIAL MESENCHYMAL TRANSITION	106	-0.34	0.371	0.504	119	-0.46	0.149	0.253
TGF BETA SIGNALING	39	-0.41	0.057	0.546	39	-0.44	0.045	0.300
TNFA SIGNALING VIA NFKB	113	-0.37	0.240	0.453	115	-0.36	0.136	0.307
PANCREAS BETA CELLS	20	-0.56	0.000	0.792	20	-0.46	0.105	0.319
APICAL JUNCTION	99	-0.31	0.317	0.491	101	-0.39	0.175	0.320
IL2 STAT5 SIGNALING	123	-0.36	0.054	0.589	122	-0.31	0.168	0.320
KRAS SIGNALING UP	106	-0.37	0.189	0.514	109	-0.38	0.191	0.331
WNT BETA CATENIN SIGNALING	29	-0.33	0.451	0.474	29	-0.38	0.197	0.354
MYOGENESIS	99	-0.39	0.202	0.461	98	-0.39	0.279	0.384
UV RESPONSE DN	108	-0.43	0.025	0.572	108	-0.42	0.124	0.395
INFLAMMATORY RESPONSE	82	-0.42	0.000	0.481	82	-0.28	0.176	0.422
ANDROGEN RESPONSE	89	-0.35	0.227	0.429	89	-0.21	0.755	0.839
FATTY ACID METABOLISM	118	-0.34	0.194	0.437				
CHOLESTEROL HOMEOSTASIS	49	-0.35	0.373	0.450	49	-0.33	0.425	0.598
**Up-Regulated States or Processes**	**0** **μg Se/g**				**0.025 μg Se/g**			
** **	**No**	**ES**	**p-value**	**q-value**	**No**	**ES**	**p-value**	**q-value**
XENOBIOTIC METABOLISM					124	0.42	0.032	0.337
IL6 JAK STAT3 SIGNALING	41	0.24	0.745	1	40	0.35	0.175	0.482
OXIDATIVE PHOSPHORYLATION					151	0.38	0.049	0.490
DNA REPAIR					106	0.31	0.325	0.500
MYC TARGETS V1					164	0.35	0.330	0.504
MTORC1 SIGNALING					157	0.35	0.177	0.533
UV RESPONSE UP					85	0.31	0.262	0.544
BILE ACID METABOLISM					72	0.35	0.145	0.548
GLYCOLYSIS					138	0.27	0.267	0.563
ADIPOGENESIS					142	0.31	0.112	0.632
HYPOXIA					141	0.24	0.458	0.652
FATTY ACID METABOLISM					118	0.34	0.144	0.678
PEROXISOME					73	0.26	0.587	0.678
HEME METABOLISM					141	0.25	0.457	0.683
ALLOGRAFT REJECTION					89	0.25	0.557	0.687

^a^Shown are the 15 Hallmark gene sets with the lowest q-values for 0 (middle) and 0.025 (right) vs. 0.4 μg Se/g for down- (top) and up-regulated (bottom) gene sets, along with number of genes found (No), enrichment score (ES) and unadjusted p-values. Also shown are selected additional gene sets.

**Fig 5:**
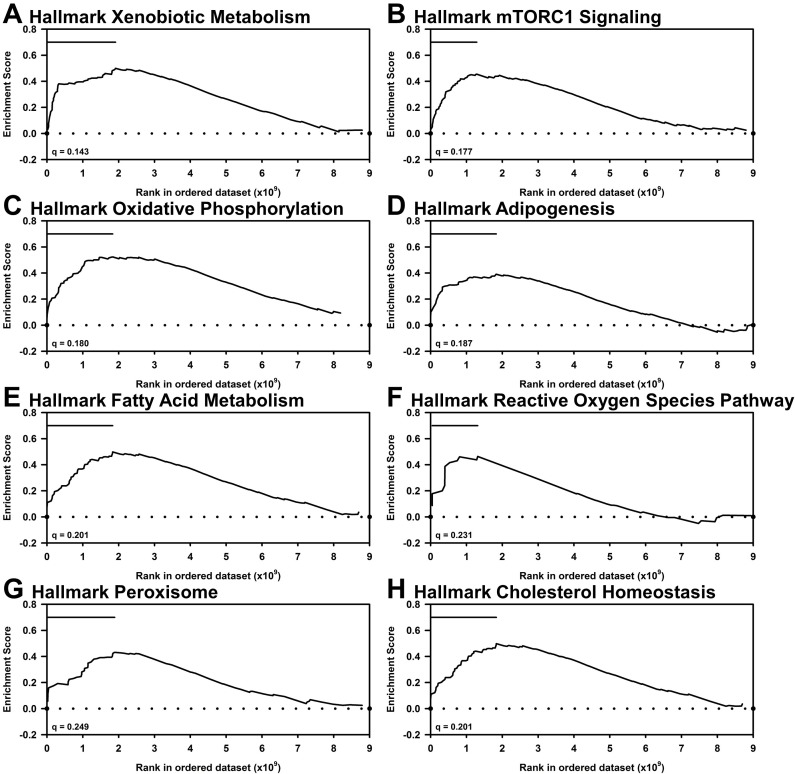
GSEA of liver transcripts in supernutritional-Se turkeys fed 1 μg Se/g vs. **0.4 μg Se/g.** Shown are resulting enrichment plots from use of the 50 Hallmark gene sets. (A) Fatty Acid Metabolism; (B) Xenobiotic Metabolism; (C) Oxidative Phosphorylation; (D) mTORC1 Signaling; (E) Adipogenesis; (F) Reactive Oxygen Species Pathway; (G) Peroxisome; (H) Cholesterol Homeostasis. Bars mark the leading-edge genes. FDR q value shown for each plot.

For high-Se supplementation, GSEA identified no Hallmark or KEGG gene sets that were significantly increased (all q > 0.9) when comparing 0.75, 2, or 5 0 μg Se/g vs. Se-adequate (0.4 μg Se/g) transcripts (**Table 5, S6 Table**). For the Hallmark gene set, only supplementation with 1 μg Se/g yielded 7 significantly up-regulated sets, including fatty acid metabolism (q = 0.201, **Fig 5**), and none of the Hallmark gene sets were significantly down-regulated by any of the four super- plus high-Se treatments (all q >0.9). Amongst KEGG gene sets, there were no significantly up-regulated sets (q > 0.25), and none were significantly down-regulated by 0.75 and 5 μg Se/g (all q >0.3, **S6 Table**). Ribosome and glyoxylate & dicarboxylate metabolism gene sets were significantly down-regulated by 2 μg Se/g (q = 0.169 and 0.182, respectively, **S2A and S2B Fig**) but not by the other Se treatments. Tight junction pathway was significantly down regulated by 1 μg Se/g (q = 0.217, **S2C Fig**) but not by the other Se treatments. Just as in Se-deficiency, GSEA found that high Se also non-significantly decreased expression of the three gene sets reported to be increased significantly by high Se in mouse liver (**S3 Fig**). Overall, GSEA did identify a very limited number of gene sets that were significantly altered by one level of super- or high-Se supplementation, but there were no pathways amongst the Hallmark or KEGG gene sets that were consistently altered by Se supplementation above Se-adequate levels.

**Table 5:** GSEA high-Se most significant Hallmark gene sets^a^.

**Down-regulated States or Processes**	**0.75 μg Se/g**				**1 μg Se/g**				**2 μg Se/g**				**5 μg Se/g**			
	**No**	**ES**	**p-value**	**q-value**	**No**	**ES**	**p-value**	**q-value**	**No**	**ES**	**p-value**	**q-value**	**No**	**ES**	**p-value**	**q-value**
ANGIOGENESIS	23	-0.48	0.156	0.811	23	-0.53	0.148	0.378	23	-0.19	0.926	0.966				
INFLAMMATORY RESPONSE	81	-0.25	0.527	0.688	84	-0.35	0.152	0.453	84	-0.23	0.646	0.891	84	-0.28	0.248	0.578
APICAL SURFACE	17	-0.23	0.972	0.922	17	-0.41	0.396	0.479	19	-0.28	0.689	0.823	18	-0.40	0.222	0.486
TNFA SIGNALING VIA NFKB	114	-0.37	0.202	0.679	116	-0.32	0.166	0.497	115	-0.25	0.577	0.800	114	-0.26	0.433	0.580
XENOBIOTIC METABOLISM	124	-0.18	0.720	0.816					124	-0.32	0.268	0.841	124	-0.31	0.187	0.500
HEDGEHOG SIGNALING	24	-0.34	0.413	0.697	22	-0.42	0.270	0.502					23	-0.42	0.051	0.789
MITOTIC SPINDLE	158	-0.32	0.298	0.690	159	-0.30	0.413	0.503	160	-0.25	0.591	0.851	160	-0.37	0.313	0.508
WNT BETA CATENIN SIGNALING	29	-0.43	0.198	0.753	29	-0.49	0.089	0.507	29	-0.28	0.736	0.790	29	-0.52	0.000	1.000
IL2 STAT5 SIGNALING	124	-0.33	0.000	0.824	123	-0.27	0.311	0.511	126	-0.26	0.294	0.827	125	-0.23	0.559	0.568
PEROXISOME	73	-0.22	0.612	0.708					74	-0.35	0.240	0.839	73	-0.37	0.206	0.512
INTERFERON ALPHA RESPONSE	51	-0.27	0.559	0.690	51	-0.33	0.309	0.569	52	-0.28	0.568	0.891	52	-0.38	0.343	0.518
INTERFERON GAMMA RESPONSE	109	-0.31	0.326	0.674	110	-0.31	0.352	0.521	112	-0.30	0.428	0.865	113	-0.34	0.099	0.602
MYC TARGETS V1	164	-0.47	0.000	0.775					164	-0.42	0.152	0.994	164	-0.39	0.407	0.529
PI3K AKT MTOR SIGNALING	72	-0.31	0.189	0.664	72	-0.27	0.490	0.570	73	-0.31	0.408	0.779	72	-0.35	0.259	0.541
ADIPOGENESIS	142	-0.27	0.235	0.665					142	-0.36	0.100	1.000	142	-0.30	0.261	0.550
PANCREAS BETA CELLS	19	-0.44	0.193	0.869	20	-0.39	0.118	0.551	19	-0.29	0.713	0.795	18	-0.36	0.335	0.585
FATTY ACID METABOLISM	118	-0.22	0.565	0.673					118	-0.37	0.167	0.716	117	-0.23	0.689	0.762
CHOLESTEROL HOMEOSTASIS	49	-0.15	0.952	1.000					49	-0.27	0.597	0.805	49	-0.14	0.970	0.985
**Up-regulated States or Processes**	**0.75 μg Se/g**				**1 μg Se/g**				**2 μg Se/g**				**5 μg Se/g**			
** **	**No**	**ES**	**p-value**	**q-value**	**No**	**ES**	**p-value**	**q-value**	**No**	**ES**	**p-value**	**q-value**	**No**	**ES**	**p-value**	**q-value**
XENOBIOTIC METABOLISM					124	0.50	0.000	0.144								
MTORC1 SIGNALING					157	0.46	0.028	0.177								
OXIDATIVE PHOSPHORYLATION					151	0.52	0.028	0.180								
ADIPOGENESIS					142	0.39	0.000	0.187								
FATTY ACID METABOLISM					118	0.50	0.000	0.201								
REACTIVE OXYGEN SPECIES PATHWAY					38	0.46	0.063	0.231								
PEROXISOME					74	0.43	0.061	0.249								
UV RESPONSE UP					85	0.39	0.026	0.278								
UNFOLDED PROTEIN RESPONSE					86	0.38	0.109	0.306								
BILE ACID METABOLISM					72	0.38	0.098	0.326								
GLYCOLYSIS					138	0.35	0.101	0.335								
HYPOXIA					125	0.30	0.136	0.416								
CHOLESTEROL HOMEOSTASIS					51	0.28	0.562	0.699								
MYC TARGETS V1					164	0.31	0.603	0.722								
ANDROGEN RESPONSE					76	0.24	0.581	0.745								
ANGIOGENESIS													23	0.16	0.970	0.947
EPITHELIAL MESENCHYMAL TRANSITION									122	0.20	0.913	1.000				
HEDGEHOG SIGNALING									24	0.22	0.837	1.000				
MYOGENESIS									101	0.11	0.978	1.000				

^a^Shown are the 15 Hallmark gene sets with the lowest q-values for 0.75, 1, 2, and 5 vs. 0.4 μg Se/g treatment, along with number of genes found (No), enrichment score (ES) and unadjusted p-values. Also shown are selected additional gene sets.

GSEA was also conducted using the total MSigDB gene ontology collection of 9996 gene sets for biological processes, cellular components, and molecular functions, comparing each dietary Se treatment vs. Se-adequate in experiment 1 and 2. GSEA GO analysis found no significant gene sets when transcripts for 0, 0.025, 0.75, 2 and 5 μg Se/g were compared to Se-adequate (lowest q values: 0.609, 0.369, 0.485, 0.475, 0.484, respectively, **S7 Table**). For 1 μg Se/g, only 2 gene sets were significant: amide transmembrane transporter activity (q = 0.243) and cellular respiration (q = 0.248). Thus GO analysis by GSEA also found virtually no processes and biological functions consistently altered by high Se. Because Se is readily metabolized by sulfur metabolism enzymes (see Discussion), the GO results from GSEA were filtered for gene sets related to sulfur or thiol metabolism. The result was 21 GO gene sets that appeared in at least 1 comparison (**S7 Table**). Except for the 1 μg Se/g transcript set, no GO gene set was significant (q > 0.490); for 1 μg Se/g, glutathione metabolic process was non-significantly up-regulated (q = 0.290) but non-significantly down-regulated for 2 and 5 μg Se/g (q ≥ 0.49). Clearly, changes in Se status did not significantly affect sulfur-related transcript expression in this study.

The GO results from GSEA were also filtered for gene sets related to oxidative stress. None of the eight oxidative stress gene sets was significant for any of the pair-wise comparison with Se-adequate treatment (all q > 0.480) (**S8 Table**).

## Discussion

### Overall gene expression effects

When Se-deficient and low-Se poults (0 and 0.025 μg Se/g) were compared to Se-adequate poults (0.4 μg Se/g), the majority of the mapped selenotranscripts were down-regulated in Se-deficiency, consistent with our previous reports [7]. These results are very similar to results in rats and mice fed Se-deficient diets as assessed using Affymetrix microarray analysis [26]. When poults fed high dietary Se (2 and 5 μg Se/g) were compared to Se-adequate poults (0.4 μg Se/g), only 2 and 14 transcripts, out of the 17,100 transcripts were DE. These small sets of DE transcript are similar in scope to the minimal effects observed when rats were fed 2 μg Se/g [16]. In the rat study, however, a 20% reduction in growth was observed when rats were fed 5 μg Se/g, which was associated with significant DE of over 1100 transcripts (4% of the transcriptome) [16]. These small sets of DE transcripts also contrast with studies in turkeys treated with a known toxin, aflatoxin, which caused differential expression of 2% of the shared transcripts [42]. In the present turkey study, where there were no signs of overt toxicity, there were few significant DE transcripts with both 2 and 5 μg Se/g, showing that the turkey poult adapts to high Se status without major changes in gene expression.

As illustrated in the volcano plots (**Fig 2**) and cluster analysis (**Fig 3**), there were few large fold differences in gene expression observed in these studies. In Se-deficiency, all significant transcript changes were < |±3|-fold. Similarly, with 5 μg Se/g, all significant transcript changes were < |±2.2|-fold. The paucity of large changes in differential gene expression further indicates that dramatic changes in gene expression do not play a role in how the turkey adapts to high Se status.

### Se-deficient and low-Se transcript expression

Se deficiency in this study resulted in significantly altering expression of 10 transcripts (0.06% of the mapped transcripts, q < 0.05), similar to what we found in rodents. Remapping against updated selenoprotein transcripts found 4 selenotranscripts significantly down-regulated by Se-deficiency (q < 0.05), with a total of 12 selenotranscripts down-regulated with a q < 0.2, similar to our previous qPCR analysis [7]. GSEA similarly found that a set of 13 selenotranscripts were significantly down-regulated in Se-deficient vs. Se-adequate liver; the selenotranscript gene set was not significantly down-regulated by 0.025 μg Se/g in GSEA. In previous studies in rats and mice, only 4 and 5 liver transcripts, respectively–all selenoproteins–were down-regulated by Se deficiency, and only 2 and 3 transcripts, respectively, were up regulated [16,26]. Clearly Se deficiency in this study results in major effects on expression of a subset but not all of the selenotranscripts, just as reported in most studies in poultry, rodents, and other species.

Analysis by DE, gene ontology, or GSEA, however, did not reveal a focused pattern of metabolic changes due to Se deficiency. The three non-selenoprotein DE transcripts down-regulated by Se-deficiency were HSPB11, an intraflagellar transport protein 25 homolog, SGTA, a small glutamine-rich tetratricopeptide repeat-containing protein alpha, and RASGRP3 (which was also significantly down-regulated by high Se); significantly up-regulated transcripts by Se-deficiency or low Se were SOCS3, a negative regulator of cytokine signaling through the JAK-STAT pathway which responds to a wide variety of stimuli [43], LOC104909734, encoding a sulfotransferase family cytosolic 2B member 1-like, LOC100545600, encoding a C-C motif chemokine 3-like protein, and BLCAP, encoding a bladder cancer associated protein (which was also significantly up-regulated by high Se). None of these transcripts were found to be associated with enriched GO pathways (**S3 Table**) or biological processes (**S4 Table**). Similarly, GSEA found no Hallmark gene sets were significantly regulated up or down by Se deficiency, and no KEGG gene sets were significantly up-regulated by Se deficiency. GSEA did yield 10 KEGG states and processes that were significantly down-regulated in Se-deficiency (q ≤ 0.25), but these gene sets were not significantly affected by 0.025 μg Se/g (**S5 Table**). GSEA did identify two Hallmark gene sets (angiogenesis and hedgehog signaling, **Table 4**) that were significantly down-regulated by 0.025 μg Se/g but not by 0 μg Se/g. These diverse states and processes, including angiogenesis, signaling, infection, disease states, and metabolic pathways, do not seem to reveal a unified change in metabolism, suggesting that these changes are more associated with an assortment of unhealthy biological conditions that are not unique to Se deficiency but rather are downstream effects due to loss of selenoprotein activity.

### High Se transcript expression

High dietary Se resulted in only small sets of 2 and 14 significant DE transcript in poults fed 2 or 5 μg Se/g in experiment 2, respectively. This is similar to what we observed in rats fed 2 μg Se/g, where only 5 and 1 transcripts were up- and down-regulated, respectively [16]; none of the genes found in rats overlap with the significantly regulated genes in the present turkey study. The turkey gene sets contain one transcript, BLCAP, which showed an apparent U-shaped response, and one transcript, RASGRP3 that showed an inverted-U-shaped response across the spectrum from Se-deficient to high-Se (**Tables 2 and 3**); each of these changes was only significant in one of the two high-Se treatments. RASGRP3 was reported to be to be up-regulated 1.65X in rats fed 5 μg Se/g diet [16], not down-regulated as in the turkey; RASGRP3 binds to dynein light chain 1 (DLC1/DYNLL1, LOC104913587 in the turkey) [44] and is an NRF2 (NFE2L2) target, suggesting that these U-shaped responses may be associated with down-stream effects of Se excess or Se deficiency, perhaps due to increased reactive oxygen species at either extreme of Se status [16]. Just as with Se-deficiency, none of the significant DE transcripts were found to be significantly enriched in GO pathways (**S3 Table**) or biological processes (**S4 Table**). Remarkably, the GSEA approach also found no Hallmark or KEGG gene sets significantly regulated by 2 or 5 μg Se/g vs. 0.4 μg Se/g in experiment 2. Thus by individual transcript DE, by GO, or by GSEA, high dietary Se had minimal effect on the hepatic turkey transcriptome, and no individual genes or pathways were found that could consistently be used as biomarkers for high Se or that suggested a role in adaptation to high Se in the turkey.

### Supernutritional Se expression

Treatment with supernutritional levels of dietary Se (0.75 and 1 μg Se/g) resulted in disparate results. With 0.75 μg Se/g, there were no significant DE transcripts in pairwise analysis relative to Se-adequate treatment. The minimal number of genes with big fold changes in the 0.75 μg Se/g group shown by cluster analysis (**Fig 3**, q < 0.1), by DE in pairwise comparison (**S2 Table**, q < 0.05), and by a lack of enriched GSEA gene sets, as well as by differences in growth and selenoenzyme expression (**Table 1**), indicate strongly that feeding 0.75 μg Se/g as selenite to the young turkey poult has no adverse effects as compared to feeding Se-adequate diets (0.4 μg Se/g).

The incremental increase from 0.75 to 1 μg Se/g in experiment 1 did affect transcript DE as there were 50 up and 38 down significant DE transcripts (**S2 Table**) with 1 vs. 0.4 μg Se/g supplementation, respectively. As discussed in Results and shown in **Table 3**, none of these transcripts were consistently altered by 2 and 5 μg Se/g, and some were also DE in the same direction by 0.025 or 0 μg Se/g (**Tables 2 and 3**), indicating that changes in DE of these transcripts were not uniquely associated with high Se status. As discussed in Results, GSEA found no significant KEGG gene sets for 1 vs 0.4 μg Se/g supplementation (**S6 Table**), but found significant increases for 6 Hallmark gene sets, including fatty acid metabolism and cholesterol homeostasis, 2 of the 3 up-regulated pathways reported in liver in mice supplemented with high Se [31]. Fatty acid metabolism, however, was non-significantly down-regulated (q >0.8) with 0.75, 2 and 5 μg Se/g treatment in this turkey study. GSEA on 1 vs 0.4 μg Se/g also found no significant down-regulated Hallmark gene sets (the pancreatic β-cell set was down-regulated non-significantly, q = 551). The only significant down-regulated KEGG gene set was the tight junction set (q = 0.217), which was not significantly regulated by 0.75, 2 or 5 μg Se/g. Thus this study did not identify one or more over-arching processes–including lipid and energy metabolism–that were consistently associated with supernutritional or high Se supplementation in turkey poults.

### Turkeys vs. rodents

As discussed above, a recent study found that GSEA identified lipid and energy metabolism as pathways in mouse liver that were significantly up-regulated by high Se (equivalent to ~3 μg Se/g diet) [31]. As reviewed above, the present turkey study provides little evidence that up-regulation of lipid and energy metabolism is associated with high Se status in the turkey. We also compared DE transcript sets for 2 and 5 μg Se/g vs. Se-adequate in the present turkey study with rat liver DE transcript sets (2 μg Se/g vs. Se-adequate) in our previous study [16]. Even when these DE transcript sets were expanded to transcript sets with unadjusted p < 0.05, there were no transcript changes associated with high Se status that were in-common between turkey poults and rats, thus providing additional support for our contention that animals do not adapt to high Se status by altering gene expression (manuscript in preparation). Reduced susceptibility to Se toxicity by the turkey as well as animal age, supplementation with high vitamin E, and duration of high Se treatment, are all likely to have contributed to the lack of common DE transcripts between rodents and turkeys in these two studies.

### Additional transcriptomics studies

A number of previous studies have assessed the effect of Se status on the full transcriptome in different species. Many of these studies, however, compared animals fed Se-adequate diets vs. Se-deficient or low-Se diets. Three of these studies in rat liver [45] and mouse liver [46,47] reported <50 significant DE transcripts q < 0.05 with selenite-supplementation vs. low Se treatment, but two studies in rat heart [48] and in chick oviduct [49] reported 4931 and 2196 transcripts, respectively, with differential expression. The cause of these elevated numbers of DE transcripts is unclear but may be due to differences in species, tissue, age of the animals, and diet including vitamin E supplementation (see below). Other Se transcriptomics studies involved other factors such as aged mice/Se-enriched rice [50], diabetic mice [51], immune challenge in trout [52], transcripts in offspring after high-fat feeding to mouse dams [53], or Se-enriched worms fed to trout [54]. In livestock, high Se supplementation as selenized yeast to cattle [55] and sheep [56] relative to Se-adequate controls reported 139 and 1186 DE transcripts in cattle liver and sheep blood, respectively. Interestingly, the cattle study used 2 different forms of Se; filtering the transcripts to those DE with both Se forms trimmed the Se-responsive set from 139 to down to 33 DE transcripts [55]. Most of the above studies only compared two treatments, whereas our study used filtering individual transcripts sets for significant transcripts DE altered in the same direction in the 5, 2 and 1 μg Se/g datasets. The result was a paucity of high-Se responsive transcripts.

### Vitamin E

Our studies in the turkey and the rat were focused on characterizing the impact of changes just due to Se status. Thus the diets used in these studies were supplemented with 12.5X (turkey) and 2X (rat) the NRC requirements for vitamin E. Older studies nicely demonstrated that the Se requirement for prevention of gizzard myopathy in turkeys was increased from 0.18 to 0.28 μg Se/g when poults were not supplemented with vitamin E [57]. In our previous rat study, feeding 5 μg Se/g resulted in 20% growth depression and significant DE of 4% of the transcriptome. A substantial number of these transcripts were NRF2 (NFE2L2) targets indicating that increased oxidative stress was associated with the effects of Se toxicity [16]. In the present turkey study, however, GSEA did not find any significant GO gene sets related to oxidative stress (**S8 Table**).

There are many studies in the literature reporting that expression of all or nearly all of the selenoprotein transcripts are significantly decreased in chickens by Se deficiency [58–59]. These studies, however, typically did not supplement the diets with vitamin E such that clinical and sub-clinical disease associated with the combined loss of Se and vitamin E likely underlie the substantial number of reported changes in gene expression. Other studies using large numbers of chicks fed Se-deficient corn-soy diets did find significant decreases in growth in Se-deficient chicks supplemented with 1X to 5X the NRC chicken vitamin E requirement [60–61]; these studies reported that 7–9 selenotranscripts were significantly decreased by Se deficiency. Supernutritional Se supplementation studies in chicks fed corn-soy diets have reported that high dietary Se decreases [62] or increases growth [63] along with increasing expression of subsets of selenotranscripts. Our studies in the young chick [18] and turkey poult [7] only found significant changes in 8 and 4 selenoprotein transcripts out of 24, respectively, in the livers of birds fed Se-deficient torula yeast diets for 4 weeks. Differences in species, diet, and vitamin E supplementation clearly contribute to differences in growth and selenotranscript expression. For the present study, diets were supplemented with elevated dietary vitamin E so that we could study the effect of high dietary Se on transcript expression without disease complications, thus focusing on the molecular effects of Se status alone.

### Other possibilities

The paucity of transcription changes associated with Se status raises several interesting alternative hypotheses. The large number of DE transcripts (**S2 Table**) and the seven significant Hallmark gene sets (**Table 5**) for 1 but not 0.75 or 2 μg Se/g might suggest that that 1 μg Se/g tweaks metabolism sufficiently to alter transcription of a number of genes, but that by 2 μg Se/g, Se metabolism readjusts to cope with the increased incoming Se. The smaller changes in liver Se concentrations in turkeys fed 0.4 to 2 μg Se/g as compared to 5 μg Se/g (**Table 1**), however, suggests that these DE transcripts are not the driving force used to adapt to additional dietary Se over this range. One attractive hypothesis is the “response to high Se” gene set is simply missing from Hallmark and KEGG gene sets; this is illustrated by GSEA using the “turkey selenoprotein” gene set which was highly significant, q = 0.023, and thus far more significant than any of the curated MSigDB gene sets. In part, these hypothetical genes involved in response to high Se may be amongst the uncharacterized LOC genes in the turkey as well as human transcriptome.

A second hypothesis is that Se is metabolized via other pathways where transcript expression is unaltered by Se status; there may have been little evolutionary pressure in mammals to enhance a pathway (gene set) to cope with high Se, perhaps mainly because Se is readily metabolized by sulfur metabolizing enzymes in animals [64] and plants [65]. Se, present at concentrations in animals over a 1000-fold less than for sulfur, simply “hitch-hikes” along these sulfur pathways, leading to formation of selenium analogs of sulfur metabolites, mixed selenodisulfides, and even a selenosugar (seleno-N-acetyl-galactosamine) in animals [66–69]. In yeast and plants, this results in synthesis of selenomethionine from inorganic Se, but this does not occur in monogastric animals [65,70,71]. In the present study, however, there was only 1 sulfur metabolism transcript with significant DE (**S2 Table**); LOC104909734, a sulfotransferase, was increased >2-fold in 0 vs. 0.4 μg Se/g, but not DE by high Se treatment. GSEA similarly did not find any significant gene ontology gene sets for 2 or 5 μg Se/g treatment related to sulfur/thiol metabolism (**S7 Table**). In other studies, we have evidence that Se accumulates in turkeys fed 5 μg Se/g as a selenosugar linked to low molecular weight thiols as well as general body proteins [71]. The pathway for formation of the selenosugar or a homologous sulfur-sugar has not been identified.

## Conclusions

These experiments studied the effect of graded levels of dietary Se fed to turkey poults in diets supplemented with vitamin E so that direct effects of altered Se status could be studied. The study affirmed that the major effect of Se-deficiency is to down-regulate expression of a subset of selenoprotein transcripts in liver, with little significant effect on general transcript expression. In response to high Se intake (2 and 5 μg Se/g) relative to Se-adequate turkeys, there were only a limited number of significant DE transcripts, all with only relatively small fold-changes; no transcript showed a consistent pattern of altered expression in response to high Se intakes across the 1, 2 and 5 μg Se/g treatments, and there were no associated metabolic pathways and biological functions that were significant and consistently found with high Se supplementation. GSEA found no gene sets that were consistently altered by high-Se and supernutritional-Se. A comparison of these DE transcript sets with those identified in high Se transcript sets parallel studies conducted in rats (2 μg Se/g) and in mice (~3 μg Se/g) fed high Se also failed to identify common DE transcripts between these three species. Collectively, this study indicates that turkeys do not alter gene expression as a homeostatic mechanism to adapt to high Se. Targeting the nature of metabolites present in liver of turkeys fed high Se has the potential to identify biomarkers for high Se status and to suggest metabolic pathways used by turkeys to adapt to high Se intake.

## Supporting information

S1 Fig GSEA of liver transcripts in turkeys fed Se-deficient vs Se-adequate diet. Shown are resulting enrichment plots (left) and fold-change heat maps (right) for resulting leading-edge genes. (A) Hallmark cholesterol homeostasis gene set; (B) Hallmark pancreatic β-cell gene set; (C) Hallmark fatty acid metabolism gene set. Enrichment score is plotted vs. the rank in the ordered dataset of 11,612 humanized turkey transcripts (trimmed to include >14 counts, and normalized). Bars marks the leading-edge genes shown in the corresponding heat map. FDR q value shown for each plot.
(PDF)

S2 Fig GSEA of liver transcripts in turkeys fed 2 or 1 μg Se/g vs. 0.4 μg Se/g. Shown are resulting enrichment plots (left) and fold-change heat maps (right) for resulting leading-edge genes. (A) KEGG ribosome gene set for 2 μg Se/g; (B) KEGG glyoxylate and dicarboxylate gene set for 2 μg Se/g; (C) KEGG tight junction gene set for 1 μg Se/g; Enrichment score is plotted vs. the rank in the ordered dataset of 11,612 humanized turkey transcripts (trimmed to include >14 counts, and normalized). Bars mark the leading-edge genes shown in the corresponding heat map. FDR q value shown for each plot.
(PDF)

S3 Fig GSEA of liver transcripts in high-Se turkeys fed 5 μg Se/g vs. 0.4 μg Se/g. Shown are resulting enrichment plots (left) and fold-change heat maps (right) for resulting leading-edge genes. (A) Hallmark cholesterol homeostasis gene set; (B) Hallmark pancreatic β-cell gene set; (C) Hallmark fatty acid metabolism gene set. Enrichment score is plotted vs. the rank in the ordered dataset of 11,612 humanized turkey transcripts (trimmed to include >14 counts, and normalized). Bar marks the leading-edge genes shown in the corresponding heat map. FDR q value shown for each plot.
(PDF)

S1 Table Gene IDs and functions of significant DE transcripts by 0, 0.025, 2, and 5 μg Se/g. (PDF)

S2 Table Transcripts differentially expressed by 0.75 and 1.0 μg Se/g vs. Se-adequate (Exp 1). (PDF)

S3 Table Enriched gene ontology pathways. (PDF)

S4 Table Enriched gene ontology biological processes. (PDF)

S5 Table GSEA Se-deficient and low-Se most significant KEGG gene sets. (PDF)

S6 Table GSEA high-Se most significant KEGG gene gets. (PDF)

S7 Table Effect of dietary Se vs. Se-adequate on GO biological processes, GO cellular components, and GO molecular functions involving sulfur or thiols, from GSEA for 9996 gene sets. (PDF)

S8 Table Effect of dietary Se vs. Se-adequate on GO biological processes, GO cellular components, and GO molecular functions involving oxidative stress, from GSEA for 9996 gene sets. (PDF)

S9 Table Human symbols raw counts. (XLSX)
