# Supplementary figures and images for: The hepatic transcriptome of the turkey poult (Meleagris gallopavo) is minimally altered by high inorganic dietary selenium
Source: PLoS One. 2020 May 7;15(5):e0232160. doi: 10.1371/journal.pone.0232160 (PMC7205448; doi:10.1371/journal.pone.0232160)

S1 Figure 1

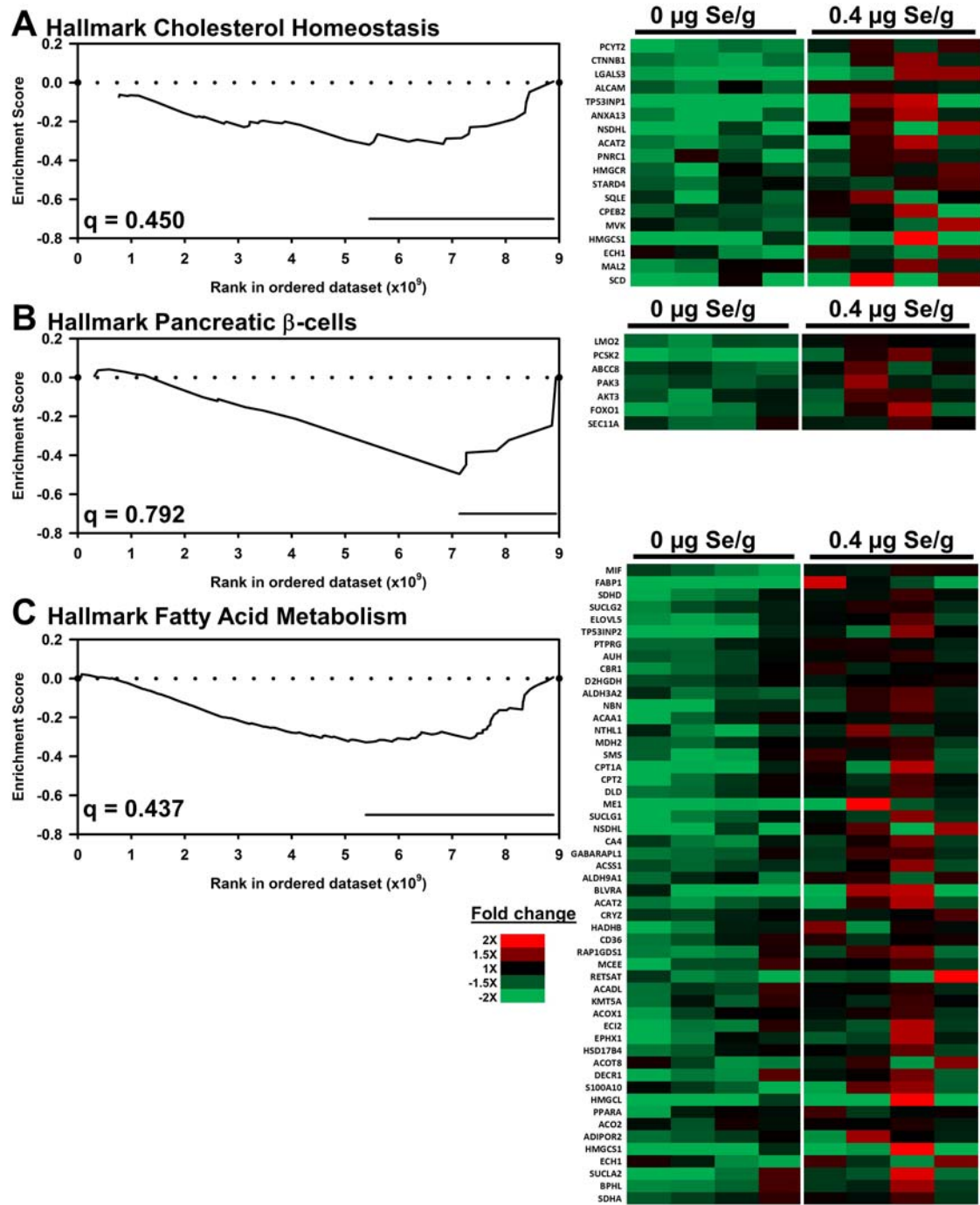

Supplement: S1 Fig — Shown are resulting enrichment plots (left) and fold-change heat maps (right) for resulting leading-edge genes. (A) Hallmark cholesterol homeostasis gene set; (B) Hallmark pancreatic β-cell gene set; (C) Hallmark fatty acid metabolism gene set. Enrichment score is plotted vs. the rank in the ordered dataset of 11,612 humanized turkey transcripts (trimmed to include >14 counts, and normalized). Bars marks the leading-edge genes shown in the corresponding heat map. FDR q value shown for each plot. (PDF) [file pone.0232160.s001.pdf]

S2 Figure 2

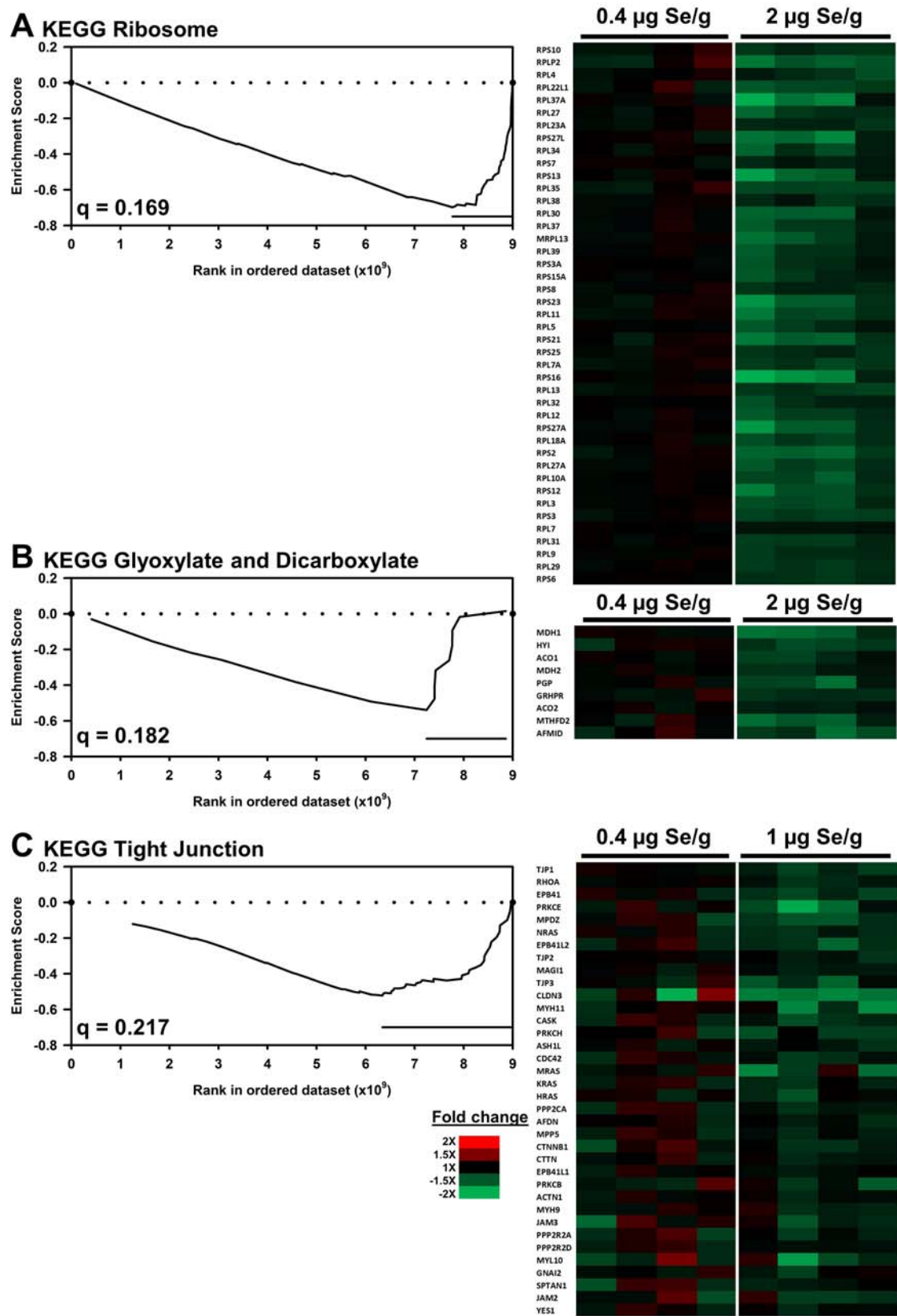

Supplement: S2 Fig — Shown are resulting enrichment plots (left) and fold-change heat maps (right) for resulting leading-edge genes. (A) KEGG ribosome gene set for 2 μg Se/g; (B) KEGG glyoxylate and dicarboxylate gene set for 2 μg Se/g; (C) KEGG tight junction gene set for 1 μg Se/g; Enrichment score is plotted vs. the rank in the ordered dataset of 11,612 humanized turkey transcripts (trimmed to include >14 counts, and normalized). Bars mark the leading-edge genes shown in the corresponding heat map. FDR q value shown for each plot. (PDF) [file pone.0232160.s002.pdf]

S3 Figure 3

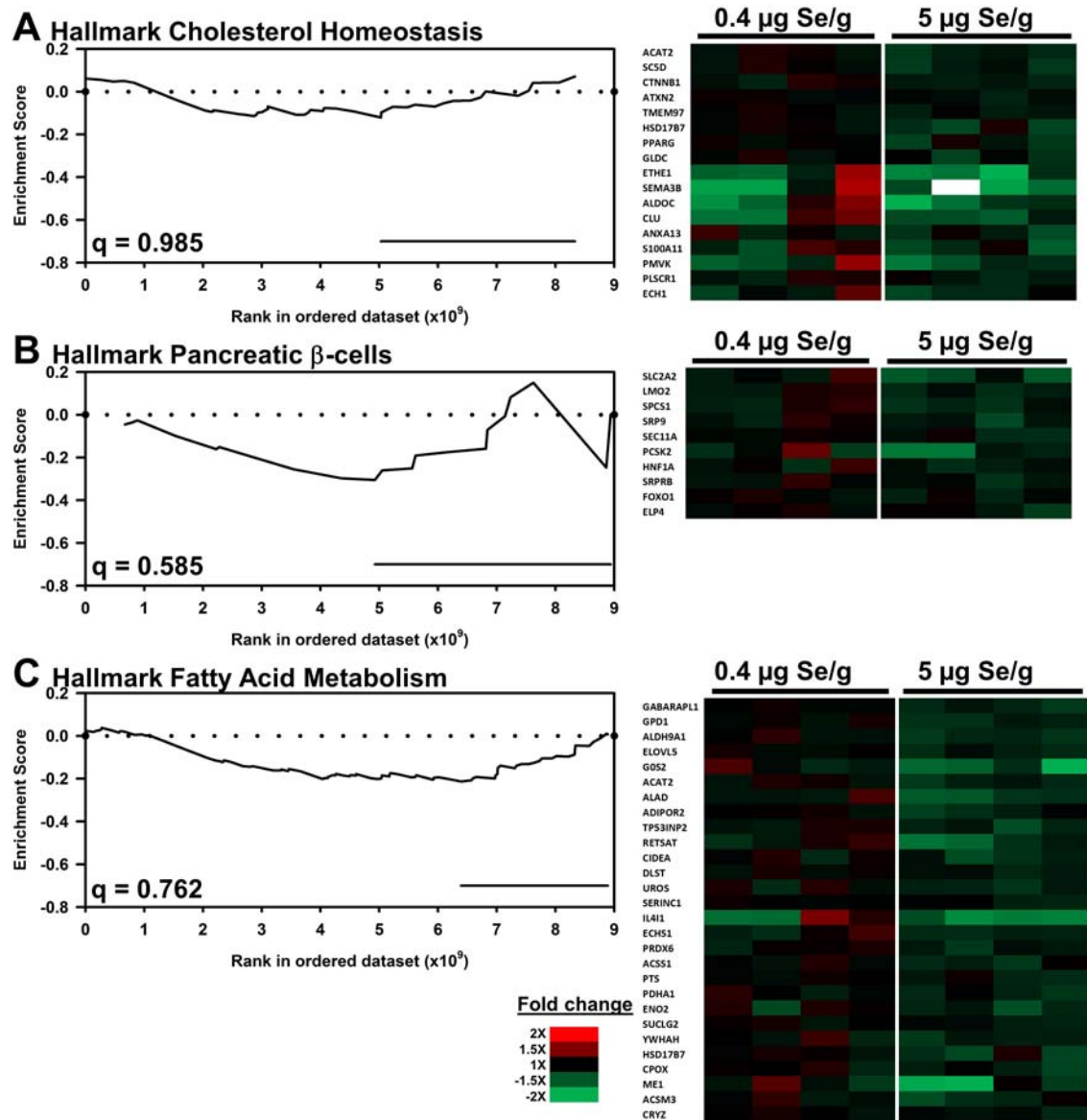

Supplement: S3 Fig — Shown are resulting enrichment plots (left) and fold-change heat maps (right) for resulting leading-edge genes. (A) Hallmark cholesterol homeostasis gene set; (B) Hallmark pancreatic β-cell gene set; (C) Hallmark fatty acid metabolism gene set. Enrichment score is plotted vs. the rank in the ordered dataset of 11,612 humanized turkey transcripts (trimmed to include >14 counts, and normalized). Bar marks the leading-edge genes shown in the corresponding heat map. FDR q value shown for each plot. (PDF) [file pone.0232160.s003.pdf]
