# Supplementary material for: The hepatic transcriptome of the turkey poult (Meleagris gallopavo) is minimally altered by high inorganic dietary selenium
Source: PLoS One. 2020 May 7;15(5):e0232160. doi: 10.1371/journal.pone.0232160 (PMC7205448; doi:10.1371/journal.pone.0232160)
Supplement: S1 Table — (PDF) [file pone.0232160.s004.pdf]

S1 Table 1. Gene IDs and functions of significant DE transcripts by 0, 0.025, 2, and 5 µg Se/g [a]

Symbol	Gene ID[b]	Dietary Se (µg Se/g)[c]	Description
ATP5G1	100548571	5	ATP synthase F(0) complex subunit C1, mitochondrial
ATPAF2	100544773	5	LOW QUALITY PROTEIN: ATP synthase mitochondrial F1 complex assembly factor 2
BLCAP	100547906	0.025, 5	bladder cancer-associated protein
BRI3	104913503	5	brain protein I3
CCND1	100539759	5	LOW QUALITY PROTEIN: G1/S-specific cyclin-D1
CDC42BPB	100550337	5	LOW QUALITY PROTEIN: serine/threonine-protein kinase MRCK beta
CHCHD2	100550053	5	coiled-coil-helix-coiled-coil-helix domain-containing protein 2
DAD1	100547759	5	dolichyl-diphosphooligosaccharide--protein glycosyltransferase subunit DAD1
DIO1	100546412	0	type I iodothyronine deiodinase deiodinase, iodothyronine type I
HSPB11	100543060	0	intraflagellar transport protein 25 homolog
LOC100545600	100545600	0	C-C motif chemokine 3-like
LOC100545745	100545745	5	cytochrome c oxidase subunit 5A, mitochondrial
LOC104909734	104909734	0	sulfotransferase family cytosolic 2B member 1-like
LOC104912775	104912775	5	PREDICTED: lipase C, hepatic type (LIPC)
PDPK1	100541724	5	3-phosphoinositide-dependent protein kinase 1
PEBP1	100545499	5	phosphatidylethanolamine-binding protein 1
RASGRP3	100539029	0, 2	ras guanyl-releasing protein 3
RNH1	100548440	5	ribonuclease inhibitor
RPS27A	100542056	5	ubiquitin-40S ribosomal protein S27a
SDPR	100545738	2	serum deprivation-response protein
SELENOP1	100547086<br>104914865	0, 0.025	selenoprotein P1
SELENOP2	100546913	0	selenoprotein P2
SELENOU	100550701	0	redox-regulatory protein FAM213A UPF0765 protein C10orf58 homolog
SGTA	100543804	0	small glutamine-rich tetratricopeptide repeat-containing alpha
SOCS3	100547594	0	suppressor of cytokine signaling 3

[a] Transcripts with significant DE (q <0.05) in pairwise comparison with Se-adequate (0.4 µg Se/g)

[b] NCBI gene ID

[c] Dietary Se level with significant DE vs. Se-adequate
