# Supplementary material for: The hepatic transcriptome of the turkey poult (Meleagris gallopavo) is minimally altered by high inorganic dietary selenium
Source: PLoS One. 2020 May 7;15(5):e0232160. doi: 10.1371/journal.pone.0232160 (PMC7205448; doi:10.1371/journal.pone.0232160)
Supplement: S2 Table — (PDF) [file pone.0232160.s005.pdf]

S2 Table2. Transcripts differentially expressed by 0.75 and 1.0 µg Se/g vs. Se-adequate (Exp 1)*

Symbol	Gene ID	Fold	p-value	q-value
**0.75 µg Se/g Differentially Expressed Transcripts**				
none				
**1.0 µg Se/g Differentially Expressed Transcripts**				
LOC104915521	104915521	3.00	2.10E-15	2.4062E-11
BLCAP	100547906	1.63	3.94E-09	1.5039E-05
LOC100540610	100540610	2.91	3.48E-09	1.5039E-05
LOC100541679	100541679	2.85	4.89E-08	1.3995E-04
ATPAF2	100544773	2.05	1.25E-07	0.0003
RNH1	100548440	1.48	2.26E-07	0.0004
PARP11	100541236	-1.58	3.10E-07	0.0005
DAD1	100547759	1.57	4.61E-07	0.0006
ATP5G1	100548571	1.64	4.18E-07	0.0006
RASGRP3	100539029	-2.58	6.35E-07	0.0007
LOC100545069	100545069	-1.68	9.60E-07	0.0010
RPS27L	104912789	1.80	1.65E-06	0.0016
PDPK1	100541724	-1.66	2.13E-06	0.0019
LOC104917166	104917166	-2.15	2.34E-06	0.0019
LOC100539914	100539914	1.60	2.63E-06	0.0020
PEBP1	100545499	1.57	4.24E-06	0.0030
LOC100549893	100549893	1.37	4.69E-06	0.0032
PHB2	100549095	1.32	5.81E-06	0.0037
DDOST	100550468	1.67	6.28E-06	0.0038
TET1	100541560	-1.74	9.03E-06	0.0052
GPD1	100542199	1.89	1.17E-05	0.0064
LOC104913587	104913587	2.02	1.60E-05	0.0083
OAZ2	100539411	1.55	1.81E-05	0.0090
PEPD	104909255	1.44	2.43E-05	0.0116
RPS14	100549126	1.67	2.70E-05	0.0120
TRIM59	100542643	1.75	2.72E-05	0.0120
ARID5B	100550037	-1.93	2.86E-05	0.0121
AARS2	100550992	1.43	3.08E-05	0.0126
AAED1	104915126	-1.73	3.18E-05	0.0126
LOC104917522	104917522	-2.16	3.77E-05	0.0144
LOC100545745	100545745	1.47	4.16E-05	0.0144
LOC104909811	104909811	2.08	4.05E-05	0.0144
LOC100541906	100541906	2.18	4.13E-05	0.0144
LOC100540238	100540238	-1.96	4.49E-05	0.0151
IQGAP1	100550295	-1.45	4.74E-05	0.0155
RPL10A	100544053	1.33	5.05E-05	0.0157
RDH10	100546900	1.85	5.06E-05	0.0157
BRI3	104913503	1.77	5.61E-05	0.0158
EEF1A1	100549103	1.32	5.67E-05	0.0158
LOC100544502	100544502	1.58	5.32E-05	0.0158
ADD3	100547330	-1.45	5.57E-05	0.0158
VRK2	100540832	-1.46	6.19E-05	0.0169
ZNF438	100539706	-1.66	6.69E-05	0.0178
RPL18A	100544743	1.33	7.37E-05	0.0181
SLC25A5	100545489	1.63	7.41E-05	0.0181
RPS2	100549328	1.45	7.22E-05	0.0181
LOC104911227	104911227	-1.85	7.43E-05	0.0181

*Fold changes, p-value, and FDR q-value for DE transcripts ($q < 0.05$) by 0.75 and 1 vs. 0.4 µg Se/g .

Continued: S2 Table. Transcripts differentially expressed by 1.0 μg Se/g vs. Se-adequate

Symbol	Gene ID	Fold	p-value	q-value
**1.0 μg Se/g Differentially Expressed Transcripts (Continued)**				
TAF4B	100542216	-2.10	7.84E-05	0.0187
USP42	104913451	-1.90	8.58E-05	0.0201
LOC100549801	100549801	1.70	9.36E-05	0.0214
LOC100539550	100539550	-1.78	9.83E-05	0.0221
RARB	100545327	-1.90	1.06E-04	0.0228
ENO1	100543180	1.52	1.05E-04	0.0228
LOC104915857	104915857	-2.00	1.13E-04	0.0236
LOC104916258	104916258	-1.99	1.12E-04	0.0236
KNOP1	104913506	-1.50	1.22E-04	0.0241
AIMP2	100543713	1.50	1.19E-04	0.0241
LOC100544389	100544389	-1.90	1.22E-04	0.0241
IL13RA2	100545119	-1.74	1.35E-04	0.0261
EVL	100546249	-1.71	1.37E-04	0.0262
SAP18	100550579	1.41	1.42E-04	0.0266
ACADS	100541626	1.42	1.46E-04	0.0270
LOC100545943	100545943	-1.93	1.57E-04	0.0284
FOXK2	100550617	-1.81	1.62E-04	0.0290
GSG2	100546729	2.05	1.70E-04	0.0299
PDCL	100547852	-1.36	1.72E-04	0.0299
THOC1	100538518	-1.31	2.09E-04	0.0347
LOC104913019	104913019	-1.50	2.05E-04	0.0347
DCAF4	100548750	1.45	2.09E-04	0.0347
QDPR	100547834	1.80	2.19E-04	0.0359
AKAP13	100551220	-1.74	2.37E-04	0.0380
LOC104910474	104910474	-1.74	2.42E-04	0.0380
CAT	100541757	1.67	2.41E-04	0.0380
TRAF3IP1	100546958	-1.84	2.48E-04	0.0383
ATF7IP	100551090	-1.44	2.51E-04	0.0384
LOC104911486	104911486	-1.74	2.58E-04	0.0389
LOC104909370	104909370	-1.94	2.69E-04	0.0395
LOC100539718	100539718	2.01	2.68E-04	0.0395
LOC100538694	100538694	1.36	2.78E-04	0.0403
TMEM204	100551175	-1.54	2.99E-04	0.0427
LOC104913034	104913034	1.75	3.09E-04	0.0427
GAPDH	100303685	1.43	3.09E-04	0.0427
GLOD4	100548719	1.53	3.06E-04	0.0427
SERHL2	100549399	1.35	3.14E-04	0.0428
LOC104912447	104912447	-1.52	3.23E-04	0.0434
RPL31	100540520	1.23	3.26E-04	0.0434
SLC30A5	100540565	1.53	3.31E-04	0.0435
LOC100541254	100541254	1.63	3.72E-04	0.0484

*Fold changes, p-value, and FDR q-value for DE transcripts (q < 0.05) by 1 vs. 0.4 μg Se/g .
