# Supplementary material for: The hepatic transcriptome of the turkey poult (Meleagris gallopavo) is minimally altered by high inorganic dietary selenium
Source: PLoS One. 2020 May 7;15(5):e0232160. doi: 10.1371/journal.pone.0232160 (PMC7205448; doi:10.1371/journal.pone.0232160)
Supplement: S3 Table — (PDF) [file pone.0232160.s006.pdf]

S3 Table 3. Enriched Gene Ontology Pathways*

Term	# DE	# Set	p-value	q-value	Genes
0 and 0.025 µg Se/g vs. Se-adequate (Exp. 1, 7 homologous transcripts)					
Selenium Metabolism and Selenoproteins WP28	2	46	0.0001	0.0509	DIO1;SELENOP
Selenium Micronutrient Network WP15	2	86	0.0004	0.0893	DIO1;SELENOP
2.0 and 5.0 µg Se/g vs. Se-adequate (Exp. 2, 12 homologous transcripts)					
Endometrial cancer WP4155	2	63	0.0006	0.2981	CCND1;PDPK1
Signaling Pathways in Glioblastoma WP2261	2	82	0.0011	0.2518	CCND1;PDPK1
Pancreatic adenocarcinoma pathway WP4263	2	89	0.0013	0.1975	CCND1;PEBP1
EGF/EGFR Signaling Pathway WP437	2	162	0.0041	0.4815	PDPK1;PEBP1
Focal Adhesion WP306	2	198	0.0060	0.5692	CCND1;PDPK1
1.0 µg Se/g vs. Se-adequate (Exp. 1, 57 homologous transcripts)					
Cytoplasmic Ribosomal Proteins WP477	5	89	5.43E-06	0.0026	RPS14;RPL18A;RPL31;RPS2;RPL10A
Mesodermal Commitment Pathway WP2857	3	147	0.0085	0.6702	ARID5B;TET1;RARB
EGF/EGFR Signaling Pathway WP437	3	162	0.0111	0.7476	PDPK1;PEBP1;IQGAP1
HIF1A and PPARG regulation of glycolysis WP2456	2	8	0.0002	0.0522	GPD1;GAPDH
Metabolic reprogramming in colon cancer WP4290	2	42	0.0064	1	ENO1;GAPDH
Vitamin A and Carotenoid Metabolism WP716	2	43	0.0067	0.7889	RDH10;RARB
Glycolysis and Gluconeogenesis WP534	2	45	0.0073	0.6894	ENO1;GAPDH
Pathways in clear cell renal cell carcinoma WP4018	2	85	0.0245	1	ENO1;GAPDH
B Cell Receptor Signaling Pathway WP23	2	97	0.0313	1	PDPK1;RASGRP3
Endoderm Differentiation WP2853	2	141	0.0612	1	TET1;TAF4B
Ciliary landscape WP4352	2	216	0.1262	1	TRAF3IP1;IQGAP1
VEGFA]VEGFR2 Signaling Pathway WP3888	2	236	0.1455	1	PDPK1;IQGAP1

*Human homologs for turkey genes with DE transcripts q < 0.05 were subjected to GO analysis using Enrichr for Human WikiPathways 2019 (https://amp.pharm.mssm.edu/Enrichr/) [39]. Gene sets were low Se (0 and 0.025 µg Se/g), moderately high Se (1 µg Se/g), and high Se (2 and 5 µg Se/g) treatments. Shown are GO term, no. of genes in set, no. of DE genes present, p-value, q-value and symbols.of DE genes present.
