# Supplementary material for: The hepatic transcriptome of the turkey poult (Meleagris gallopavo) is minimally altered by high inorganic dietary selenium
Source: PLoS One. 2020 May 7;15(5):e0232160. doi: 10.1371/journal.pone.0232160 (PMC7205448; doi:10.1371/journal.pone.0232160)
Supplement: S4 Table — (PDF) [file pone.0232160.s007.pdf]

S4 Table 4. Enriched Gene Ontology Biological Processes*

Term	# DE	# Set	p-value	q-value	Genes
0 and 0.025 µg Se/g vs. Se-adequate (Exp. 1, 7 homologous transcripts)					
none with 2 or more differentially expressed transcripts)					
2.0 and 5.0 µg Se/g vs. Se-adequate (Exp. 2, 12 homologous transcripts)					
cellular protein modification process (GO:0006464)	3	1002	0.0196	1	DAD1;PDPK1;CDC42BPB
regulation of transcription from RNA polymerase II promoter (GO:0006357)	3	1479	0.0536	1	CHCHD2;CCND1;RPS27A
negative regulation of cellular response to transforming growth factor beta stimulus (GO:1903845)	2	54	0.0005	1	PDPK1;RPS27A
negative regulation of transforming growth factor beta receptor signaling pathway (GO:0030512)	2	56	0.0005	1	PDPK1;RPS27A
negative regulation of transmembrane receptor protein serine/threonine kinase signaling pathway (GO:0090101)	2	78	0.0010	1	PDPK1;RPS27A
regulation of transforming growth factor beta receptor signaling pathway (GO:0017015)	2	80	0.0010	1	PDPK1;RPS27A
ERBB signaling pathway (GO:0038127)	2	81	0.0010	1	PDPK1;RPS27A
stimulatory C-type lectin receptor signaling pathway (GO:0002223)	2	122	0.0023	1	PDPK1;RPS27A
innate immune response activating cell surface receptor signaling pathway (GO:0002220)	2	125	0.0025	1	PDPK1;RPS27A
regulation of G2/M transition of mitotic cell cycle (GO:0010389)	2	150	0.0035	1	CCND1;RPS27A
T cell receptor signaling pathway (GO:0050852)	2	164	0.0042	1	PDPK1;RPS27A
Fc-epsilon receptor signaling pathway (GO:0038095)	2	183	0.0052	1	PDPK1;RPS27A
Fc receptor signaling pathway (GO:0038093)	2	184	0.0052	1	PDPK1;RPS27A
regulation of I-kappaB kinase/NF-kappaB signaling (GO:0043122)	2	205	0.0064	1	PDPK1;RPS27A
mitotic cell cycle phase transition (GO:0044772)	2	222	0.0075	1	CCND1;RPS27A
activation of protein kinase activity (GO:0032147)	2	234	0.0083	1	PDPK1;RPS27A
antigen receptor-mediated signaling pathway (GO:0050851)	2	258	0.0100	1	PDPK1;RPS27A
MAPK cascade (GO:0000165)	2	279	0.0117	1	PEBP1;RPS27A
phosphorylation (GO:0016310)	2	387	0.0217	1	PDPK1;CDC42BPB
regulation of intracellular signal transduction (GO:1902531)	2	423	0.0256	1	PDPK1;RPS27A
cellular response to cytokine stimulus (GO:0071345)	2	457	0.0296	1	CCND1;RPS27A
protein phosphorylation (GO:0006468)	2	471	0.0312	1	PDPK1;CDC42BPB
negative regulation of transcription from RNA polymerase II promoter (GO:0000122)	2	566	0.0437	1	CCND1;RPS27A
cytokine-mediated signaling pathway (GO:0019221)	2	634	0.0537	1	CCND1;RPS27A
1.0 µg Se/g vs. Se-adequate (Exp. 1, 57 homologous transcripts)					
peptide biosynthetic process (GO:0043043)	6	175	1.03E-05	0.0105	EEF1A1;RPS14;RPL18A;RPL31;RPS2;RPL10A
protein targeting to ER (GO:0045047)	5	98	8.72E-06	0.0111	RPS14;RPL18A;RPL31;RPS2;RPL10A
viral transcription (GO:0019083)	5	114	1.82E-05	0.0116	RPS14;RPL18A;RPL31;RPS2;RPL10A
cotranslational protein targeting to membrane (GO:0006613)	5	94	7.11E-06	0.0121	RPS14;RPL18A;RPL31;RPS2;RPL10A
nuclear-transcribed mRNA catabolic process, nonsense-mediated decay (GO:0000184)	5	113	1.74E-05	0.0127	RPS14;RPL18A;RPL31;RPS2;RPL10A
viral gene expression (GO:0019080)	5	111	1.60E-05	0.0136	RPS14;RPL18A;RPL31;RPS2;RPL10A
SRP-dependent cotranslational protein targeting to membrane (GO:0006614)	5	90	5.74E-06	0.0147	RPS14;RPL18A;RPL31;RPS2;RPL10A
translation (GO:0006412)	7	233	4.29E-06	0.0219	EEF1A1;RPS14;AIMP2;RPL18A;RPL31;RPS2;RPL10A
cellular response to epidermal growth factor stimulus (GO:0071364)	3	26	0.0001	0.0309	EEF1A1;PDPK1;IQGAP1
nuclear-transcribed mRNA catabolic process (GO:0000956)	5	175	0.0001	0.0716	RPS14;RPL18A;RPL31;RPS2;RPL10A
gene expression (GO:0010467)	7	412	0.0002	0.0755	EEF1A1;RPS14;RPL18A;RPL31;THOC1;RPS2;RPL10A
rRNA processing (GO:0006364)	5	203	0.0003	0.1098	RPS14;RPL18A;RPL31;RPS2;RPL10A
rRNA metabolic process (GO:0016072)	5	201	0.0003	0.1136	RPS14;RPL18A;RPL31;RPS2;RPL10A
viral process (GO:0016032)	5	221	0.0004	0.1506	RPS14;RPL18A;RPL31;RPS2;RPL10A
ncRNA processing (GO:0034470)	5	228	0.0005	0.1519	RPS14;RPL18A;RPL31;RPS2;RPL10A
cytoplasmic translation (GO:0002181)	3	55	0.0005	0.1556	RPL18A;RPL31;RPL10A
ribosome biogenesis (GO:0042254)	5	227	0.0005	0.1588	RPS14;RPL18A;RPL31;RPS2;RPL10A
cellular macromolecule biosynthetic process (GO:0034645)	6	368	0.0006	0.1738	EEF1A1;RPS14;RPL18A;RPL31;RPS2;RPL10A
ribosomal small subunit assembly (GO:0000028)	2	19	0.0013	0.3553	RPS14;RPS27L
hepaticobiliary system development (GO:0061008)	2	21	0.0016	0.4130	ARID5B;RARB
cellular protein metabolic process (GO:0044267)	6	485	0.0025	0.5344	EEF1A1;RPS14;RPL18A;RPL31;RPS2;RPL10A
glucose catabolic process to pyruvate (GO:0061718)	2	26	0.0025	0.5507	ENO1;GAPDH
canonical glycolysis (GO:0061621)	2	26	0.0025	0.5757	ENO1;GAPDH
glycolytic process through glucose-6-phosphate (GO:0061620)	2	26	0.0025	0.6031	ENO1;GAPDH
protein alkylation (GO:0008213)	2	30	0.0033	0.6732	EEF1A1;RPS2
liver development (GO:0001889)	2	32	0.0037	0.7354	ARID5B;RARB
bone development (GO:0060348)	2	35	0.0045	0.8448	AKAP13;RARB
erythrocyte differentiation (GO:0030218)	2	36	0.0047	0.8610	RPS14;ATPIF1

*Human homologs for turkey genes with DE transcripts q < 0.05 were subjected to GO analysis using Enrichr for Human Biological Process 2018 (https://amp.pharm.mssm.edu/Enrichr/) [39]. Gene sets were low Se (0 and 0.025 µg Se/g), moderately high Se (1 µg Se/g), and high Se (2 and 5 µg Se/g) treatments. Shown are GO term, no. of genes in set, no. of DE genes present, p-value, q-value and symbols.of DE genes present,
