# Supplementary material for: The hepatic transcriptome of the turkey poult (Meleagris gallopavo) is minimally altered by high inorganic dietary selenium
Source: PLoS One. 2020 May 7;15(5):e0232160. doi: 10.1371/journal.pone.0232160 (PMC7205448; doi:10.1371/journal.pone.0232160)
Supplement: S5 Table — (PDF) [file pone.0232160.s008.pdf]

S5 Table 5. GSEA Se-deficient and low-Se most significant KEGG gene sets*

Down-Regulated States or Processes	0 µg Se/g				0.025 µg Se/g			
	No	ES	p-value	q-value	No	ES	p-value	q-value
LEISHMANIA INFECTION	29	-0.64	0	0.085	30	-0.4	0.103	0.377
BASAL TRANSCRIPTION FACTORS	24	-0.62	0	0.201	24	-0.4	0.196	0.462
WNT SIGNALING PATHWAY	86	-0.45	0	0.214	84	-0.4	0.065	0.525
HUNTINGTONS DISEASE	111	-0.47	0.054	0.215				
ARACHIDONIC ACID METABOLISM	17	-0.62	0	0.222	17	-0.41	0.251	0.395
GAP JUNCTION	46	-0.47	0	0.227	47	-0.45	0	0.488
ALZHEIMERS DISEASE	92	-0.45	0.02	0.232				
LEUKOCYTE TRANSENDOTHELIAL MIGRATION	62	-0.48	0.063	0.232	61	-0.48	0.042	0.525
FC EPSILON RI SIGNALING PATHWAY	43	-0.48	0	0.233	43	-0.3	0.579	0.642
CALCIUM SIGNALING PATHWAY	87	-0.39	0	0.25	86	-0.24	0.463	0.564
NATURAL KILLER CELL MEDIATED CYTOTOXICITY	50	-0.49	0	0.273	49	-0.45	0.042	0.42
CELL ADHESION MOLECULES CAMS	34	-0.48	0	0.274	35	-0.44	0.109	0.433
T CELL RECEPTOR SIGNALING PATHWAY	61	-0.49	0	0.277	60	-0.49	0.031	0.587
CYTOSOLIC DNA SENSING PATHWAY	21	-0.53	0	0.279				
TIGHT JUNCTION	65	-0.44	0.028	0.281	65	-0.42	0.131	0.349
FATTY ACID METABOLISM	27	-0.44	0.247	0.436				
INSULIN SIGNALING PATHWAY	92	-0.28	0.346	0.458				
CYSTEINE AND METHIONINE METABOLISM	25	-0.31	0.567	0.55				
**Up-Regulated States or Processes**	**0 µg Se/g**				**0.025 µg Se/g**			
	No	ES	p-value	q-value	No	ES	p-value	q-value
PENTOSE PHOSPHATE PATHWAY	16	0.35	0.486	1	16	0.68	0	0.234
ETHER LIPID METABOLISM					43	0.59	0	0.249
RIBOSOME					70	0.63	0.031	0.263
CYSTEINE AND METHIONINE METABOLISM					25	0.56	0	0.297
HISTIDINE METABOLISM					16	0.5	0.172	0.472
GLYCEROPHOSPHOLIPID METABOLISM					45	0.47	0.057	0.476
HUNTINGTONS DISEASE					111	0.32	0.09	0.48
OXIDATIVE PHOSPHORYLATION					66	0.42	0.064	0.489
PARKINSONS DISEASE					69	0.38	0.122	0.503
SELENOAMINO ACID METABOLISM	15	0.34	0.621	1	15	0.52	0.12	0.511
GLYOXYLATE AND DICARBOXYLATE METABOLISM					16	0.44	0.126	0.514
RNA POLYMERASE					21	0.52	0.116	0.532
SNARE INTERACTIONS IN VESICULAR TRANSPORT					27	0.46	0.161	0.532
ONE CARBON POOL BY FOLATE					15	0.45	0.193	0.536
GLUTATHIONE METABOLISM	29	0.3	0.306	1	29	0.41	0.124	0.54
FATTY ACID METABOLISM					27	0.41	0.412	0.728
BIOSYNTHESIS OF UNSATURATED FATTY ACIDS					16	0.33	0.603	0.762

*Shown are the 15 KEGG gene sets with the lowest q-values for 0 and 0.025 vs. 0.4 µg Se/g treatment for down- and up-regulated gene sets, along with number of genes found (No), enrichment score (ES), and unadjusted p-values. Also shown are selected additional gene sets.
