# Supplementary material for: The hepatic transcriptome of the turkey poult (Meleagris gallopavo) is minimally altered by high inorganic dietary selenium
Source: PLoS One. 2020 May 7;15(5):e0232160. doi: 10.1371/journal.pone.0232160 (PMC7205448; doi:10.1371/journal.pone.0232160)
Supplement: S6 Table — (PDF) [file pone.0232160.s009.pdf]

S6 Table 6. High-Se GSEA most significant KEGG Gene Sets*

Down-regulated States or Processes	0.75 µg Se/g				1 µg Se/g				2 µg Se/g				5 µg Se/g			
	No	ES	p-value	q-value	No	ES	p-value	q-value	No	ES	p-value	q-value	No	ES	p-value	q-value
RIBOSOME	70	-0.32	0.582	0.701					70	-0.71	0	0.169	70	-0.35	0.676	0.691
GLYOXYLATE AND DICARBOXYLATE METABOLISM	16	-0.45	0.158	0.483					15	-0.62	0.016	0.182	15	-0.32	0.58	0.675
TIGHT JUNCTION	65	-0.46	0	0.731	65	-0.53	0	0.217	65	-0.21	0.873	0.812	64	-0.33	0.358	0.549
DORSO VENTRAL AXIS FORMATION	17	-0.46	0.019	0.668	17	-0.47	0.11	0.295	17	-0.27	0.8	0.777	17	-0.45	0.167	0.416
ACUTE MYELOID LEUKEMIA	35	-0.49	0	0.827	35	-0.47	0	0.303	36	-0.26	0.681	0.769	36	-0.42	0.15	0.404
ERBB SIGNALING PATHWAY	60	-0.38	0.106	0.441	60	-0.4	0.061	0.313	59	-0.26	0.71	0.768	59	-0.42	0.134	0.462
AXON GUIDANCE	75	-0.4	0.229	0.468	73	-0.44	0.117	0.314	77	-0.15	0.933	0.988	74	-0.29	0.522	0.674
NEUROTROPHIN SIGNALING PATHWAY	88	-0.32	0.199	0.516	88	-0.44	0.089	0.325	89	-0.3	0.39	0.74	87	-0.43	0.05	0.387
VEGF SIGNALING PATHWAY	45	-0.38	0.138	0.48	45	-0.44	0.137	0.325	47	-0.35	0.258	0.85	46	-0.45	0.027	0.349
FC EPSILON RI SIGNALING PATHWAY	43	-0.43	0.119	0.5	43	-0.48	0.169	0.328	44	-0.4	0.045	0.661	44	-0.43	0.024	0.387
ENDOMETRIAL CANCER	35	-0.52	0	1	35	-0.56	0	0.352	36	-0.39	0.133	0.728	36	-0.54	0	0.33
THYROID CANCER	20	-0.58	0	0.616	20	-0.51	0.04	0.33	20	-0.45	0.039	0.636	20	-0.49	0.052	0.416
MELANOGENESIS	50	-0.38	0.118	0.446	50	-0.4	0.108	0.336	49	-0.22	0.83	0.81	50	-0.38	0.247	0.409
GAP JUNCTION	48	-0.4	0.133	0.459	48	-0.44	0.098	0.338	47	-0.23	0.879	0.809	45	-0.39	0.115	0.448
REGULATION OF ACTIN CYTOSKELETON	123	-0.4	0.1	0.529	123	-0.4	0.025	0.338	123	-0.28	0.468	0.684	121	-0.35	0.045	0.398
CYSTEINE AND METHIONINE METABOLISM	25	-0.33	0.342	0.593					25	-0.45	0.17	0.611	25	-0.36	0.31	0.543
SELENOAMINO ACID METABOLISM	15	-0.41	0.407	0.579					15	-0.36	0.475	0.717	15	-0.29	0.735	0.696
GLUTATHIONE METABOLISM	29	-0.2	0.849	0.817					29	-0.32	0.401	0.729	29	-0.28	0.664	0.717
FATTY ACID METABOLISM	27	-0.23	0.756	0.82					27	-0.46	0.315	0.84	27	-0.27	0.822	0.875
**Up-regulated States or Processes**	**0.75 µg Se/g**				**1 µg Se/g**				**2 µg Se/g**				**5 µg Se/g**			
	No	ES	p-value	q-value	No	ES	p-value	q-value	No	ES	p-value	q-value	No	ES	p-value	q-value
ONE CARBON POOL BY FOLATE					15	0.56	0.039	0.252								
ARGININE AND PROLINE METABOLISM					34	0.51	0.02	0.262								
OXIDATIVE PHOSPHORYLATION					66	0.53	0.066	0.269								
BUTANOATE METABOLISM					23	0.56	0.053	0.286								
PENTOSE PHOSPHATE PATHWAY	16	0.17	0.975	1	16	0.7	0	0.295								
VALINE LEUCINE AND ISOLEUCINE DEGRADATION					37	0.55	0.057	0.308								
PEROXISOME					64	0.47	0	0.349								
SELENOAMINO ACID METABOLISM					15	0.51	0.163	0.351								
GLUTATHIONE METABOLISM					30	0.62	0	0.355								
GLYOXYLATE AND DICARBOXYLATE METABOLISM					16	0.55	0.042	0.356								
TRYPTOPHAN METABOLISM					26	0.47	0.051	0.356								
PROTEIN EXPORT					23	0.51	0.218	0.361								
PYRUVATE METABOLISM					29	0.45	0.2	0.366								
N GLYCAN BIOSYNTHESIS					36	0.41	0.174	0.368								
CYSTEINE AND METHIONINE METABOLISM					25	0.37	0.172	0.371								
GLYCOLYSIS GLUCONEOGENESIS	33	0.13	0.911	0.98	34	0.53	0.104	0.402								
GLYCINE SERINE AND THREONINE METABOLISM	22	0.2	0.918	1	22	0.51	0.461	0.626								
HEMATOPOIETIC CELL LINEAGE	21	0.24	0.821	1	21	0.35	0.352	0.672								
PPAR SIGNALING PATHWAY					40	0.33	0.556	0.739					40	0.14	0.961	0.987
DILATED CARDIOMYOPATHY									27	0.16	0.969	0.977	29	0.19	0.939	1
ECM RECEPTOR INTERACTION					45	0.16	0.905	0.99	46	0.2	0.88	1				
HYPERTROPHIC CARDIOMYOPATHY HCM													28	0.17	0.935	1

*Shown are the 15 KEGG gene sets with the lowest q-values for 0.75, 1, 2, and 5 vs. 0.4 µg Se/g treatment, along with number of genes found (No), enrichment score (ES) and unadjusted p-values. Also shown are selected additional gene sets.
