# Supplementary material for: The hepatic transcriptome of the turkey poult (Meleagris gallopavo) is minimally altered by high inorganic dietary selenium
Source: PLoS One. 2020 May 7;15(5):e0232160. doi: 10.1371/journal.pone.0232160 (PMC7205448; doi:10.1371/journal.pone.0232160)
Supplement: S7 Table — (PDF) [file pone.0232160.s010.pdf]

S7 Table. Effect of dietary Se vs. Se-adequate on GO biological processes, GO cellular components, and GO molecular functions involving sulfur or thiols, from GSEA for 9996 gene sets

	0 µg Se/g		0.025 µg Se/g		0.75 µg Se/g		1 µg Se/g		2 µg Se/g		5 µg Se/g	
**GSEA GO Process**[a]	**NESb**[b]	**q-value**[c]	**NES**	**q-value**	**NES**	**q-value**	**NES**	**q-value**	**NES**	**q-value**	**NES**	**q-value**
2 IRON 2 SULFUR CLUSTER BINDING	0.71	0.855	0.87	0.992	-1.25	0.512	0.97	0.864	-1.35	0.787	-1.10	0.512
4 IRON 4 SULFUR CLUSTER BINDING	-0.90	1.000	1.18	1.000	-1.36	0.553	1.40	0.514	-1.38	0.793	-0.97	0.579
ACID THIOL LIGASE ACTIVITY	-0.99	1.000	0.47	0.996	-0.76	0.771	0.62	0.955	-1.10	0.734	-0.85	0.690
CHONDROITIN SULFATE BIOSYNTHETIC PROCESS	-0.69	1.000	-1.15	0.617	-0.67	0.867	-0.71	0.858	-0.87	0.798	-0.97	0.585
CHONDROITIN SULFATE PROTEOGLYCAN BIOSYNTHETIC PROCESS	-0.69	1.000	-1.10	0.651	-0.70	0.829	0.56	0.974	-0.87	0.798	-0.95	0.594
CHONDROITIN SULFATE PROTEOGLYCAN METABOLIC PROCESS	0.60	0.939	-1.16	0.617	-0.69	0.847	0.50	0.984	-0.78	0.840	-0.88	0.657
DISULFIDE OXIDOREDUCTASE ACTIVITY	1.13	0.617	1.08	1.000	-1.09	0.535	1.12	0.802	-1.16	0.737	-1.11	0.508
GLUTATHIONE METABOLIC PROCESS	1.25	0.652	1.26	1.000	0.76	1.000	1.61	0.290	-0.76	0.846	-0.49	0.983
HEPARAN SULFATE PROTEOGLYCAN METABOLIC PROCESS	-0.62	1.000	-0.78	0.857	-0.92	0.629	0.63	0.948	-0.97	0.762	-1.18	0.490
INTRASPECIES INTERACTION BETWEEN ORGANISMS											-0.85	0.690
IRON SULFUR CLUSTER ASSEMBLY	1.00	0.634	1.25	1.000	-0.88	0.662	1.19	0.739	-1.22	0.765	-1.23	0.499
KERATAN SULFATE BIOSYNTHETIC PROCESS	1.28	0.693	-1.28	0.548			-0.73	0.847	0.50	1.000	-0.75	0.792
LIGASE ACTIVITY FORMING CARBON SULFUR BONDS	-0.92	1.000	0.68	0.982	-0.80	0.741	0.97	0.868	-1.19	0.741	-0.82	0.716
OXIDOREDUCTASE ACTIVITY ACTING ON A SULFUR GROUP OF DONORS	1.01	0.630	1.17	1.000	-1.23	0.508	1.34	0.592	-1.31	0.784	-1.26	0.503
PROTEIN DISULFIDE ISOMERASE ACTIVITY							1.25	0.692				
PROTEIN DISULFIDE OXIDOREDUCTASE ACTIVITY			-0.77	0.859	-1.08	0.537	-0.97	0.670	-1.27	0.779	-1.35	0.542
S ADENOSYLMETHIONINE DEPENDENT METHYLTRANSFERASE ACTIVITY	1.29	0.724	1.22	1.000	-1.58	0.553	-0.89	0.721	-1.37	0.791	-1.18	0.490
SULFOTRANSFERASE ACTIVITY	1.15	0.615	-0.98	0.719	-0.80	0.742	-0.59	0.938	-0.46	0.986	-1.18	0.490
SULFUR COMPOUND BINDING	0.68	0.880	-1.25	0.566	-1.28	0.522	-0.98	0.663	-0.66	0.901	-1.02	0.551
SULFUR COMPOUND BIOSYNTHETIC PROCESS	0.84	0.747	1.01	1.000			1.30	0.633	-1.12	0.738	-0.90	0.647
SULFUR COMPOUND CATABOLIC PROCESS	0.94	0.681	-1.03	0.689	-0.88	0.664	0.74	0.912	-1.10	0.737	-0.77	0.771
SULFUR COMPOUND METABOLIC PROCESS	0.90	0.700	1.04	1.000	-0.98	0.576	1.22	0.706	-1.10	0.740	-0.85	0.690
SULFUR COMPOUND TRANSMEMBRANE TRANSPORTER ACTIVITY	1.18	0.619	1.34	0.984	-1.01	0.565	1.30	0.634	-0.69	0.882	-0.91	0.625
SULFUR COMPOUND TRANSPORT	1.09	0.614	1.29	1.000	-0.97	0.589	1.29	0.657	-0.68	0.891	-1.02	0.551
THIOESTER BIOSYNTHETIC PROCESS	-0.53	1.000	1.07	1.000	-0.57	0.935	1.55	0.352	-1.04	0.742	-0.83	0.713
THIOESTER METABOLIC PROCESS	-0.62	1.000	-0.65	0.933	-0.75	0.780	0.87	0.867	-1.16	0.734	-0.82	0.716
THIOLESTER HYDROLASE ACTIVITY	0.89	0.703	1.10	1.000	-0.82	0.718	1.37	0.551	-1.20	0.747	-1.27	0.507
TRANSFERASE ACTIVITY TRANSFERRING SULFUR CONTAINING GROUPS	0.96	0.659	1.13	1.000	-0.82	0.718	1.10	0.815	-1.24	0.771	-1.37	0.548
**Minimum q-value:**		**0.614**		**0.548**		**0.508**		**0.290**		**0.734**		**0.490**

[a] GO process present in at least one comparison with Se-adequate (0.4 µg Se/g)

[b] Normalized enrichment score (NES). Negatitive sign (-) indicates down-direction of regulation of process by indicated Se treatment

[c] FDR q-value
