# Supplementary material for: The hepatic transcriptome of the turkey poult (Meleagris gallopavo) is minimally altered by high inorganic dietary selenium
Source: PLoS One. 2020 May 7;15(5):e0232160. doi: 10.1371/journal.pone.0232160 (PMC7205448; doi:10.1371/journal.pone.0232160)
Supplement: S8 Table — (PDF) [file pone.0232160.s011.pdf]

S8 Table. Effect of dietary Se vs. Se-adequate on GO biological processes, GO cellular components, and GO molecular functions involving oxidative stress, from GSEA for 9996 gene sets

GSEA GO Process[a]	0 µg Se/g		0.025 µg Se/g		0.75 µg Se/g		1 µg Se/g		2 µg Se/g		5 µg Se/g	
	NESb[b]	q-value[c]	NES	q-value	NES	q-value	NES	q-value	NES	q-value	NES	q-value
CELL DEATH IN RESPONSE TO OXIDATIVE STRESS	1.19	0.601	0.68	0.991	-1.18	0.495	0.83	0.911	-1.30	0.768	-1.36	0.574
CELLULAR RESPONSE TO OXIDATIVE STRESS	0.82	0.755	0.90	1.000	-1.12	0.513	0.93	0.908	-1.12	0.715	-1.25	0.505
INTRINSIC APOPTOTIC SIGNALING PATHWAY IN RESPONSE TO OXIDATIVE STRESS	0.80	0.770	-0.63	0.948	-1.17	0.494	0.90	0.900	-1.55	0.669	-1.48	0.739
NEGATIVE REGULATION OF OXIDATIVE STRESS INDUCED INTRINSIC APOPTOTIC SIGNALING PATHWAY	1.22	0.610	-1.13	0.591	-1.28	0.490	-1.05	0.617	-1.45	0.759	-1.33	0.545
NEGATIVE REGULATION OF RESPONSE TO OXIDATIVE STRESS	1.22	0.610	-0.73	0.894	-1.19	0.492	0.76	0.928	-1.24	0.744	-1.14	0.487
NEURON DEATH IN RESPONSE TO OXIDATIVE STRESS	1.33	0.712	-0.64	0.941	-1.07	0.538	0.79	0.921	-1.52	0.691	-1.67	1.000
REGULATION OF OXIDATIVE STRESS INDUCED CELL DEATH	1.42	0.835	-0.90	0.767	-1.30	0.497	-0.80	0.786	-1.14	0.722	-1.25	0.506
REGULATION OF OXIDATIVE STRESS INDUCED INTRINSIC APOPTOTIC SIGNALING PATHWAY	1.27	0.637	-1.10	0.619	-1.35	0.501	-0.92	0.695	-1.38	0.738	-1.42	0.638
REGULATION OF RESPONSE TO OXIDATIVE STRESS	1.21	0.612	-0.81	0.832	-1.25	0.484	0.70	0.935	-1.17	0.710	-1.38	0.584
RESPONSE TO OXIDATIVE STRESS	0.92	0.675	0.81	0.999	-1.24	0.482	1.01	0.906	-1.13	0.719	-1.28	0.518
**Minimum q-value:**		**0.601**		**0.591**		**0.482**		**0.617**		**0.669**		**0.487**

[a] GO process present in at least one comparison with Se-adequate (0.4 µg Se/g)

[b] Normalized enrichment score (NES). Negatitive sign (-) indicates down-direction of regulation of process by indicated Se treatment

[c] FDR q-value
